# Impact of spatial proximity on territoriality among human skin bacteria

**DOI:** 10.1038/s41522-020-00140-0

**Published:** 2020-08-06

**Authors:** Jhonatan A. Hernandez-Valdes, Lu Zhou, Marcel P. de Vries, Oscar P. Kuipers

**Affiliations:** 1 Department of Molecular Genetics, Groningen Biomolecular Sciences and Biotechnology Institute, University of Groningen, Nijenborgh 7, 9747 AG Groningen, The Netherlands; 2 Department of Biomedical Engineering Antonius Deusinglaan 1, University Medical Center Groningen, Groningen University, 9713 AW Groningen, Netherlands

**Keywords:** Microbial ecology, Microbiome

## Abstract

Bacteria display social behavior and establish cooperative or competitive interactions in the niches they occupy. The human skin is a densely populated environment where many bacterial species live. Thus, bacterial inhabitants are expected to find a balance in these interactions, which eventually defines their spatial distribution and the composition of our skin microbiota. Unraveling the physiological basis of the interactions between bacterial species in organized environments requires reductionist analyses using functionally relevant species. Here, we study the interaction between two members of our skin microbiota, *Bacillus subtilis* and *Staphylococcus epidermidis*. We show that *B. subtilis* actively responds to the presence of *S. epidermidis* in its proximity by two strategies: antimicrobial production and development of a subpopulation with migratory response. The initial response of *B. subtilis* is production of chlorotetain, which degrades the *S. epidermidis* at the colony level. Next, a subpopulation of *B. subtilis* motile cells emerges. Remarkably this subpopulation slides towards the remaining *S. epidermidis* colony and engulfs it. A slow response back from *S. epidermidis* cells give origin to resistant cells that prevent both attacks from *B. subtilis*. We hypothesized that this niche conquering and back-down response from *B. subtilis* and *S. epidermidis*, respectively, which resembles other conflicts in nature as the ones observed in animals, may play a role in defining the presence of certain bacterial species in the specific microenvironments that these bacteria occupy on our skin.

## Introduction

Diverse populations of human-associated bacteria live on the skin and are harmless or beneficial to their human host^1^. Symbiotic bacteria occupy skin niches and protect against colonization by pathogenic bacteria^2^. For example, *Bacillus subtilis* is known to protect the skin by producing bacitracin, a compound that inhibits the growth of other bacteria^3,4^. Although extensive research on the influence of the skin bacteria in the skin health has been performed^5,6^, few studies have investigated the specific interactions between members of the skin microbiota. The elucidation of these interactions is necessary to understand the complex organization of microbial communities e.g., the human microbiota.

Since the skin surface is a nutrient-poor niche^1^, microbial species are likely to compete for resources and colonization. The outcome of this microbial competition is the delicate balance in the diversity of bacterial species that inhabit our skin^7,8^. Bacteria employ different chemical and physical mechanisms to harm, inhibit or kill their competitors^9^. Chemical mechanisms include secretion of broad-spectrum antibiotics^10^ or strain-specific bacteriocins^11^, whereas physical mechanisms such as adhesion^12^ or secretion of polymeric substances^13^ allow bacteria to interfere with the growth of others. Here we study the potential interactions among *Bacillus subtilis* and *Staphylococcus epidermidis*. Both bacterial species are inhabitants of the same ecological niche, the human skin.

*B. subtilis* is a relevant member of the human microbiota. This bacterium produces and secretes several molecules to control the growth of other bacteria^14^, probably as an adaptive strategy to subsist in the densely populated environments where it is found (skin, digestive tract, extremities of human body and soil)^7,15,16^. *B. subtilis* is the second most frequently associated bacteria in neonates^17^. In addition, in healthy individuals (including adults) it has been found in the outer ear skin^17^, and it is predominately found in the plantar skin of humans with strong foot odor^18^. *S. epidermidis* is a major inhabitant of the skin, and comprises more than 90% of the aerobic resident microbiota^2^. Despite the innocuous nature of *S. epidermidis*, this bacterium is currently seen as an important opportunistic pathogen in patients receiving medical devices^19^. *S. epidermidis* is one of the most frequent nosocomial infections, and in particular it is involved in the infection of catheters and implants^19,20^.

Recent studies show that bacteria form biofilms in response to ecological competition^21^. In this respect, *Bacillus subtilis* is a master in cell differentiation and several subpopulations of functionally distinct cell types coexist within its biofilms^22^. Besides biofilm formation, *B. subtilis* is a model for motility of Gram-positive bacteria; it is able to swim, swarm and slide^23,24^. The use of flagella allows the bacterium to swim and swarm, but the flagellum-independent sliding occurs due to growth^25^. Sliding is a poorly understood process, but it is known to depend upon multiple factors, such as the production of surfactin and/or the extracellular proteins BslA and TasA^25–27^.

Here, we study the *B. subtilis* antimicrobial production and sliding motility in response to the presence of *S. epidermidis*. Bacterial colonies are able to interact at a close proximity, and by using a colony model we simulate the interactions between bacterial communities. Firstly, we start our analysis by visualizing the *B. subtilis* production of antimicrobials, which inhibit the growth of *S. epidermidis*. Cells of the attacked *S. epidermidis* colony rapidly develop resistance and give rise to resistant colonies. The traditional approach to study antimicrobials is based on the extraction of the compounds from culture supernatants, followed by screening of fractions to identify the inhibitory molecules^28^. Bacterial competition approaches, where two bacteria are co-cultured, allow us to revisit the mechanisms involved in competitive interactions between bacteria^29^. In this respect, genomic DNA sequencing of *S. epidermidis* resistant colonies revealed a point-nonsense mutation in the gene encoding the di-peptide transport system (DtpT) and several mutations in the gene encoding the biofilm-associated protein (Bap). Interestingly, bacilysin, the simplest peptide antibiotic known, is such a dipeptide synthesized by *B. subtilis*^30^. We demonstrate that *B. subtilis* produces chlorotetain, a halogenated variant of bacilysin, which kills *S. epidermidis* cells. Secondly, after the chemical attack, motile *B. subtilis* emerges from the colony to dramatically engulf the wild-type *S. epidermidis* colony. This migratory response is not developed towards the *S. epidermidis* resistant colonies. Therefore, our results suggest that functional Bap proteins indirectly trigger the motility of *B. subtilis*. Lastly, we show that the response regulator DegU regulates the behavior phenomena exhibited by *B. subtilis*, and it appears to mediate the interaction between *B. subtilis* and *S. epidermidis*.

## Results

### Interaction between *B. subtilis* and *S. epidermidis* colonies

**Fig. 1:**
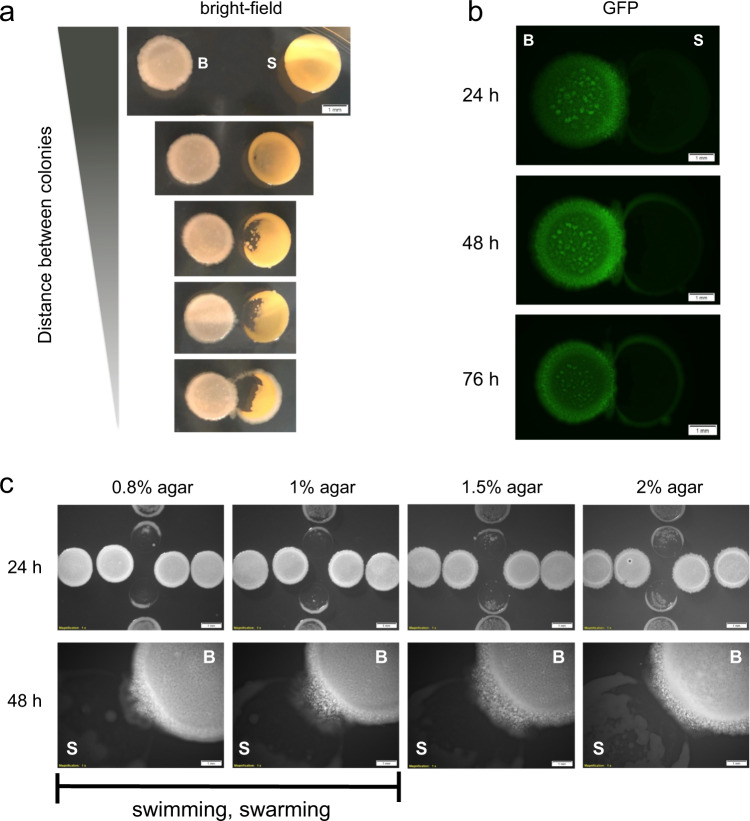
*B. subtilis* antimicrobial production and sliding in the proximity of *S. epidermidis*. **a** Interaction assay of bacterial colonies of *B. subtilis* (B) and *S. epidermidis* (S) at different separation distances on CDM-agar plates, after 28 h incubation at 37 °C. Snapshots of microscope observations bright field are shown. Scale bar, 1 mm. **b** Interaction between bacterial colonies at different incubation times. A *B. subtilis* GFP+ (B) and *S. epidermidis* GFP− (S) are shown. Snapshots of observations by fluorescence microscopy are shown. Scale bar, 1 mm. **c**, *B. subtilis* motility in CDM-agar plates with varying concentrations of agar (0.8, 1, 1.5, and 2%). Observations after 24 h incubation (top images) of a T-shape interaction assay where *B. subtilis* (colonies located at the horizontal line) and *S. epidermidis* (colonies located at the vertical line) are shown. Observations after 48 h incubation highlight the *B*. *subtilis* (B) motility towards *S. epidermidis* (S). Snapshots of observations by fluorescence microscopy are shown. Scale bar, 1 mm.

We start by analyzing whether *B. subtilis* and *S. epidermidis* are able to interact at the colony level, using a chemically defined medium (CDM) to simulate the interactions among bacterial communities in poor-nutrient niches. To assess the interaction between colonies, both bacteria are spotted on a CDM-agar plate at different separation distances. This interaction assay shows that the colonies are able to interact at a close proximity, resulting in growth inhibition of *S. epidermidis* (Fig. 1). The shorter the distance between colonies, the higher the growth inhibition effect on the *S. epidermidis* colony. Notably, *B. subtilis* develops an active migratory response towards *S. epidermidis*. At the closest proximity, *B. subtilis* cells are able to engulf the colony of *S. epidermidis*. The growth inhibition and migratory response phenomena are not observed when each species is independently grown (Fig. S1). Moreover, we tested the interaction between *B. subtilis* with another skin bacterium (*Cutibacterium brevis*) and with other Gram-positive bacteria (*Lactococcus lactis and Streptococcus thermophilus*), and noted that the colony degradation only occurred against *S. epidermidis* (Fig. S2a). We observed the colony engulfment capacity of *B. subtilis* to other bacteria, as reported in a previous study on the interactions between different *Bacillus* species^31^. Interestingly, in an interaction assay between *B. subtilis*, *S. epidermidis* and *L. lactis* (Fig. S2b), *B. subtilis* specifically responds only to the presence of *S. epidermidis*. This result suggests that only *B. subtilis* and *S. epidermidis* appear to establish this specific interaction (antimicrobial production and motility) at the colony level.

Next, we examine the time it takes to observe both responses. To distinguish the growth of *B. subtilis* from *S. epidermidis*, we used a green fluorescent protein (GFP)-marked *B. subtilis* strain. Figure 1b shows that the inhibitory growth effect occurs rapidly and is clearly visible at the end of 24 h of incubation, whereas the *B. subtilis* motility is a progressive response completed after 48 to 72 h of incubation. To this end, we aimed to distinguish the *B. subtilis* motility strategy. Swimming, swarming and sliding are the three motility strategies employed by *B. subtilis*^32^. Since an agar concentration below 1.5% (w/v) in CDM-agar plates allow *B. subtilis* to swim and swarm, Fig. 1c shows that 48 h of incubation, *B. subtilis* is able to display motility towards *S. epidermidis* at even an agar concentration of 2% (w/v). With respect to the growth inhibition effect on *S. epidermidis*, the varying concentrations of agar in the growth medium did not change the inhibitory effect. These findings suggest that *B. subtilis* is able to attack *S. epidermidis* in two phases, the first phase involves antimicrobial production, and the second phase consists of mobilization of cells via sliding to engulf the neighbor colony.

### Genetic elements involved in the *B. subtilis* sliding response

*B. subtilis* motility has been extensively studied, and its transcriptional and post-transcriptional regulation have been described in detail^23,25,32^. The master regulator DegU, known to control the production of extracellular enzymes such as the bacillopeptidase (encoded by the *bpr* gene)^33^, also coordinates motility^34^, colony architecture^35^ and cell differentiation^36^. Biofilm formation, and flagellum and non-flagellum dependent motility are regulated by the transcription factor SinR^37^. The *B. subtilis* biofilm consists of cells attached to each other by an extracellular matrix, and the production of the matrix occurs in cells with inhibited flagellar motility^22^. The flagellar filament protein that provides the origin to the flagellum is encoded by the *hag* gene, and the extracellular matrix production is encoded by the *epsE-O* operon to produce exopolysaccharides (EPS) while the major protein component of the matrix, TasA, is encoded by the *yqxM-sipW-tasA* operon^38^. Despite the fact that sliding motility does not depend on a flagellum, it does depend on surfactin (encoded by the *srfA* gene), EPS, and extracellular proteins such as the biofilm-surface layer protein A (BslA) and TasA^25^.

**Fig. 2:**
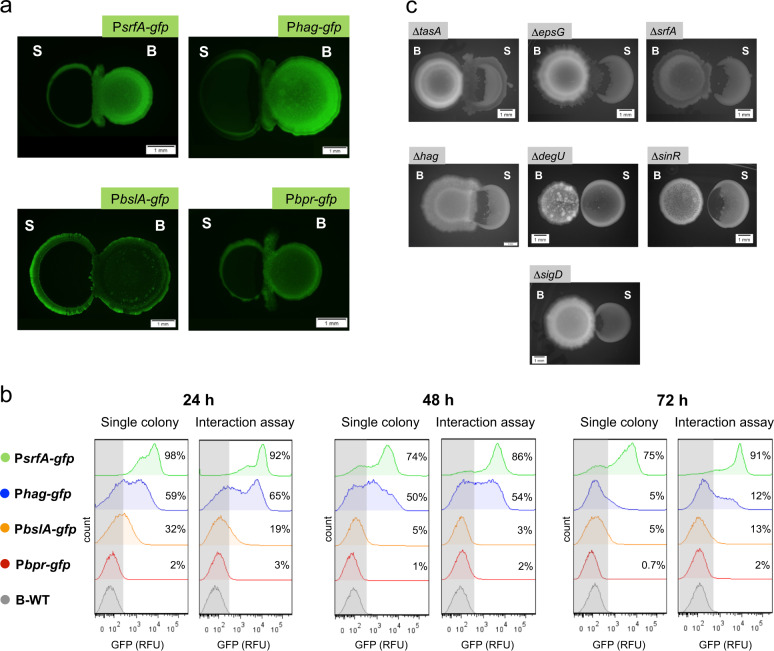
Genetic elements of *B. subtilis* involved on its antimicrobial and motility response. **a***B. subtilis* (B) strains bearing transcription fusions of promoters (Ps*rfA*, P*hag*, P*bslA*, P*bpr*) with the gene encoding the green fluorescent protein (*gfp*) in interaction assays against *S. epidermidis* (S). Snapshots of colonies on CDM-agar plates incubated at 37 °C for 72 h. Scale bar, 1 mm. **b** Single-cell fluorescence measurements by flow cytometry, in the *B. subtilis* (B) strains bearing transcription fusions of promoters (Ps*rfA*, P*hag*, P*bslA*, P*bpr*) with the gene encoding the green fluorescent protein (*gfp*) in single colonies (left) and interaction assays against *S. epidermidis* (S; right), at different incubation times (24, 48, and 72 h). The percentage of cells with higher gene expression above the background fluorescence level (above the B-WT fluorescence; indicated with a gray window) is shown. 30,000 ungated events for each sample are shown. **c***B. subtilis* deletion mutants (*tasA, epsG, srfA, hag, degU*, and *sinR*) in in interaction assays against *S. epidermidis (S)*. Snapshots of colonies on CDM-agar plates incubated at 37 °C for 48 h. Scale bar, 1 mm.

To identify the genetic requirements for motility, we aimed to test whether a specific process, e.g., motility, matrix production or surfactin production occurs in the *B. subtilis* migratory cells during the interaction assay. To this end, we evaluated the green fluorescent protein (GFP) expression in colonies of our interaction assay. We used *B. subtilis* strains bearing transcriptional fusions of several gene promoters with the *gfp* gene. Figure 2a shows that both the migratory cells and the *B. subtilis* colony activate the promoters controlling the expression of surfactin (*srfA*), flagellin (*hag*), bacillopeptidase (*brp*) and the BslA protein (*bslA*). Therefore, we investigated their expression at the single-cell level, after dispersing the biofilms, by using flow cytometry (see “Methods” section). Figure 2b shows the percentages of cells with *srfA*, *hag*, *brp*, *bslA* expression, compared to the *B. subtilis* wild type (B-WT), as single colonies (left) and in the interaction assays against *S. epidermidis* (right), at three sampling points (24, 48, and 72 h). The single-cell measurements allowed us to identify two highly activated subpopulations: motile *B. subtilis* cells (*hag*) and surfactin-secreting (*srfA*) cells (Fig. S3). This result indicates that these *B. subtilis* subpopulations underline the bacterial interaction against *S. epidermidis*. For instance, we observe that at an early incubation time (24 h), *hag* is highly expressed (65% in the interaction assay), whereas at late incubation time (72 h), surfactin (*srfA*, highly expressed in 91% cells of the interaction assay) plays an important role. Our data is in agreement with the observation of complete engulfment of the *S. epidermidis* colony after 72 h incubation in our interaction assays.

Next, we tested the migratory response of *B. subtilis* deletion mutants of key genes involved in motility, matrix production and motility. Figure 2b shows the effect of strains affected in the production of the extracellular matrix (TasA, EPS) on motility and antimicrobial production. A slight defect in migratory response for the *epsG* mutant, and no motility defects are observed for the *tasA* mutant. With respect to surfactin, the deletion of *srfA* results in a low motility response. Remarkably, the *hag* mutant shows a reduced migratory response (Fig. 2c and Fig. S4), and since sliding represents a flagellum-independent motility, this result confirms that *B. subtilis* employs sliding motility in our interaction assays. We also evaluated the deletion mutants of the main regulators DegU and SinR. Importantly, the lack of DegU results in a lack of antimicrobial production and absence of the migratory response (Fig. 2c and S5). In contrast, the lack of SinR results in antimicrobial production, but no migratory response. Previous studies conclude that SinR is a regulator that controls the transition between motile and non-motile cells in order to form multicellular communities^39^.

As noted in Fig. 2a, the production of the extracellular enzyme bacillopeptidase (*bpr*) does not play a role in this bacterial interaction. We further studied the role of extracellular enzymes by using *B. subtilis* WB800, an eight protease-deficient strain (*nprE*, *nprB*, *aprE*, *epr*, *bpr, mpr*, *vpr*, *wprA*)^40^. Fig. S6 shows that *B. subtilis* WB800 is able to degrade the *S. epidermidis* colony. Therefore, we discarded the possibility that the extracellular proteases are responsible for the *S. epidermidis* colony degradation.

Together, these findings show that *B. subtilis* develops sliding motility, involving motile cells (*hag*) and surfactin-expressing cells (*srfA*), to reach and surround *S. epidermidis*. The regulator SinR coordinates this process. In addition, the master regulator DegU not only participates in controlling the motility response, but also in the antimicrobial production against *S. epidermidis*.

### Development of antimicrobial resistance in *S. epidermidis*

**Fig. 3:**
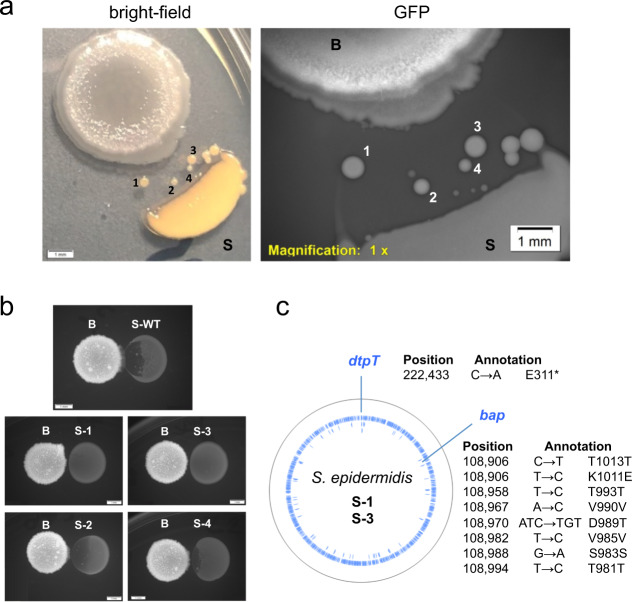
*S. epidermidis* resistant colonies emerge. **a** Interaction assay of *B. subtilis* (B) and *S. epidermidis* (S) colonies on CDM-agar plates incubated at 37 °C for 6 days. Resistant colonies selected are indicated with numbers (1 to 4). Snapshots of observations with bright-field (left) and fluorescence (right) microscopy are shown. Scale bar, 1 mm. **b** Interaction assay with *B. subtilis* wild type strain (B) and *S. epidermidis* wild type (S-WT) and selected mutants (S-1, S-2, S-3, and S-4). Snapshots of observations with fluorescence microscopy are shown. Scale bar, 1 mm. **c** Mutations in the genomic sequence of both *S. epidermidis* S-1 and S-3 strains. Location and description of mutations in the *dtpT* and *bap* genes are indicated.

Next, we performed an interaction assay where *B. subtilis* and *S. epidermidis* colonies have a separation distance that does not allow *B. subtilis* to slide and reach the neighbor colony, but it does allow the antimicrobial compound produced by *B. subtilis* to diffuse and degrade (part of) the neighbor colony. Resistant cells of *S. epidermidis* emerge and give rise to bacterial colonies after six days of incubation. Figure 3a shows several resistant colonies, and four of them (indicated with numbers) were selected to gain insight into the nature of the antimicrobial compound secreted by *B. subtilis*. Next, we confirmed the resistance of the selected *S. epidermidis* strains in an interaction assay against *B. subtilis*. Figure 3b shows that the highest antimicrobial resistance is observed in the strains 1 (S-1) and 3 (S-3), whereas the strains 2 (S-2) and 4 (S-4) are partially resistant compared to the wild type (S-WT). To investigate whether genetic mutations are responsible of the resistant phenotype, the genomes of S-1 and S-3 were sequenced. The genomic sequence data reveals that these strains have mutations located in only two coding genes, i.e., a point non-sense mutation in the *dtpT* gene encoding the dipeptide transport system DtpT and several mutations in the *bap* gene encoding the biofilm-associated protein Bap. Remarkably, the mutation in *dtpT* causes a non functional dipeptide transport system. Therefore, this finding suggests that a dipeptide with antimicrobial activity is produced by *B. subtilis* against *S. epidermidis*.

### Chlorotetain production and genetic regulation

**Fig. 4:**
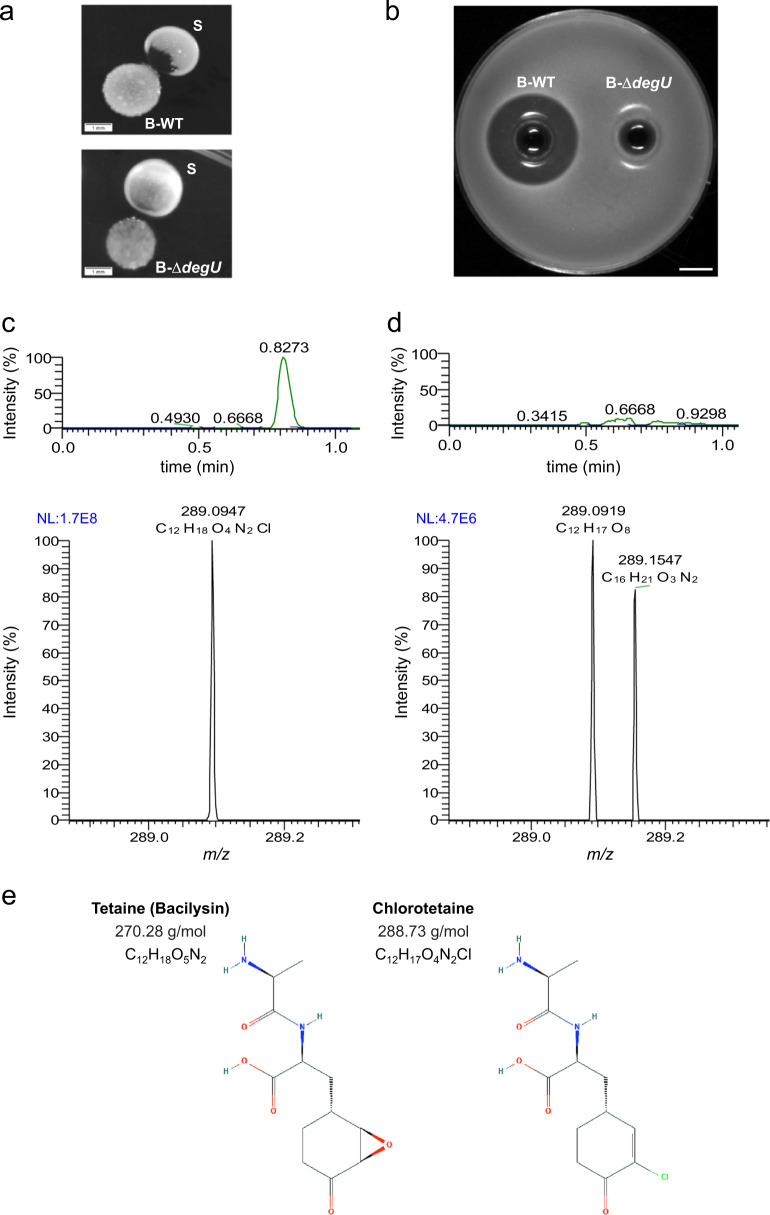
Chlorotetain production by *B. subtilis*. **a** Interaction assay between *B. subtilis* wild type (B-WT) and *S. epidermidis* wild type (S) strains (top image), and between *B. subtilis degU* deletion mutant (B-Δ*degU*) and *S. epidermidis* wild type (S) strains (bottom image). Snapshots of colonies on CDM-agar plates incubated at 37 °C for 24 h. Scale bar, 1 mm. **b** Antimicrobial activity of overnight bacterial supernatants of *B. subtilis* wild type (B-WT; left) and *B. subtilis degU* deletion mutant (B-Δ*degU*) strains on CDM-agar plates. *S. epidermidis* wild type strain was used as indicator strain. A snapshot of the antimicrobial activity, indicated by the *S. epidermidis* growth inhibition halo, was taken after incubation at 37 °C for 24 h. Scale bar, 15 mm. **c**, **d** UHPLC chromatograms (top, *x*-axis indicates time in minutes, and *y*-axis indicates percentage of signal intensity) and mass spectra (bottom, *x*-axis indicates m/z, mass-to-charge ratio; and *y*-axis indicates percentage of signal intensity), respectively, to confirm the presence of chlorotetain in overnight bacterial supernatants of *B. subtilis* wild type (**c**) and *B. subtilis degU* deletion mutant **d**. The molecular weight of the peak of the mass spectrum in **c** corresponds to chlorotetain. **e**, chemical structures of bacilysin (left) and chlorotetain (right), the molecular weight and chemical formula is indicated.

Previous studies on non-ribosomal peptides (NRPs) produced by *B. subtilis* have shown that this bacterium is able to secrete bacilysin (also known as tetaine) and its chlorinated derivatives bromotetain and chlorotetain^14^. Bacilysin is the simplest antibiotic known, made up of L-alanine and L-anticapsin^30^. This dipeptide is active against bacteria and some fungi like *Candida albicans*^41^. It is transported into the host cells, and its hydrolyzation by peptidases releases the L-anticapsin, which inhibits the glucosamine 6-phosphate (GlcN6P) synthase, resulting in cell death^30,42^. Interestingly, DegU was shown to positively regulate the biosynthesis of bacilysin in *Bacillus amyloliquefaciens*^43^. DegU and the DegS kinase are a two component signal system that controls several cellular processes, including motility and biofilm formation^44^. Thus, we tested a *degU* deletion mutant in the interaction assay against *S. epidermidis*. Figure 4a shows that the lack of DegU results in very low levels of *S. epidermidis* colony degradation, compared to the interaction with wild-type *B. subtilis*. Moreover, overnight *B. subtilis* bacterial supernatants of both wild type and Δ*degU* strains were tested for antimicrobial production using *S. epidermidis* as indicator strain (Fig. 4b). Accordingly, the supernatant of the Δ*degU* strain shows no antimicrobial activity. We performed ultra high-performance liquid chromatography coupled with mass spectrometry (UHPLC-M) to identify the chemical structure of the antimicrobial compound. The differential analysis of the compounds present in both bacterial supernatants (wild type and Δ*degU*; Fig. 4c, d, respectively) clearly shows that the wild-type strain secretes the bacilysin derivative, chlorotetain (Fig. 4c). This result is in contrast to the supernatant of the Δ*degU* strain, which does not contain chlorotetain (Fig. 4d). Chlorotetain is detected at 0.8273 min with *m*/*z* = 289.09 ([*M* + H]^+^).

**Fig. 5:**
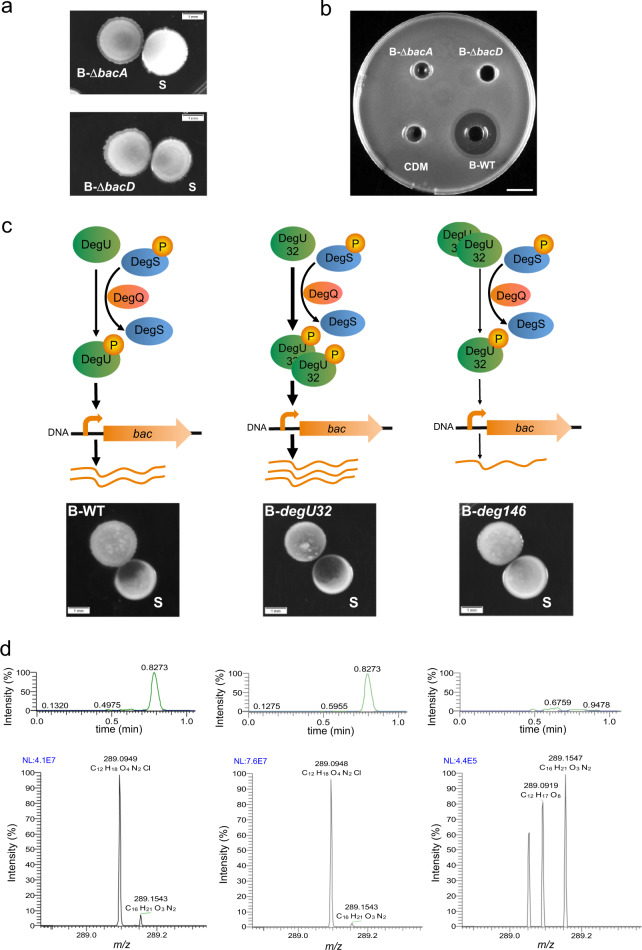
Genetic elements involved in chlorotetain production by *B. subtilis*. **a** Interaction assay between bacilysin *B. subtilis* mutants (B-Δ*bacA* and B-Δ*bacD*) with the wild-type *S. epidermidis* (S) strain, top and bottom images, respectively. Snapshots of colonies on CDM-agar plates incubated at 37 °C for 24 h. Scale bar, 1 mm. **b** Antimicrobial activity of overnight bacterial supernatants of the bacilysin *B. subtilis* mutant (B-Δ*bacA* and B-Δ*bacD*) and wild-type *B. subtilis* (B-WT) strains on CDM-agar plates. *S. epidermidis* wild-type strain was used as indicator strain. CDM, used for bacterial growth, is shown as a negative control. Snapshot of the antimicrobial activity, indicated by the *S. epidermidis* growth inhibition halo, was taken after incubation at 37 °C for 24 h. Scale bar, 10 mm. **c** Proposed model (top) of the relationship between the phosphorylation of DegU (highlighted with different thickness in the arrow), and its effect on bacilysin biosynthesis. Corresponding interaction assays (bottom) between *B. subtilis degU* mutants (B-*degU32* and B-*degU146;* center and right, respectively) strains targeting the DegU phosphorylation levels and *S. epidermidis* wild type (S) strain. An interaction assay between wild-type strains of *B. subtilis* (B-WT) and *S. epidermidis* (S) was used as a control (left). Snapshots of colonies on CDM-agar plates incubated at 37 °C for 24 h. Scale bar, 1 mm. **d**, UHPLC chromatograms (top, *x*-axis indicates time in minutes, and *y*-axis indicates percentage of signal intensity) and mass spectra (bottom, *x*-axis indicates m/z, mass-to-charge ratio; and *y*-axis indicates percentage of signal intensity), respectively, to confirm the concentration levels of chlorotetain in overnight bacterial supernatants of *B. subtilis degU* mutants (B-*degU32* and B-*degU146;* center and right, respectively) compared to the *B. subtilis* wild type (left). The molecular weight (289.09 g mol^−1^) of the peaks of the mass spectra corresponds to chlorotetain.

The enzymes participating in the synthesis of bacilysin are encoded in the *bacABCDE* (*ywfBCDEF*) gene cluster of *B. subtilis*^45^. We aimed to confirm that bacilysin is solely responsible for the *S. epidermidis* colony degradation. To this end, we performed an interaction assay with two bacilysin mutant strains (*B. subtilis* Δ*bacA* and Δ*bacD)*. Figure 5a shows that the bacilysin mutants are unable to degrade the *S. epidermidis* colony. In addition, the bacterial supernatant of any of bacilysin mutants shows no antimicrobial activity compared to the growth inhibition by the supernatant of the wild-type *B. subtilis* (B-WT) strain (Fig. 5b). Altogether, these results confirm that chlorotetain is solely responsible for the *S. epidermidis* colony degradation.

The gene regulation by DegU is dependent on its phosphorylation (DegU-P) by the DegS kinase^36^. Both forms of DegU, phosphorylated and non-phosphorylated, control different cellular processes. For instance the extracellular production of degradative enzymes requires high levels of DegU-P^44^. A previous study on bacilysin biosynthesis by *B. amyloliquefaciens* shows that both DegU and DegU-P are able to bind the *bacABCDE* operon promoter to produce bacilysin^43^. Based on this evidence, we next analyzed the contribution of DegU and DegU-P to the production of bacilysin in vivo. To achieve this aim, we used two *B. subtilis* strains with different phosphorylation levels of DegU. The *degU32* mutant has been reported to show high levels of the phosphorylated form of DegU (DegU-P), whereas the *degU146* mutant shows low levels of DegU-P^46^. We used these *degU* mutant strains in the interaction assay. Figure 5c shows increased bacilysin production by the *B. subtilis degU32* mutant and reduced bacilysin production by the *degU146* mutant. In addition, we identified the presence and concentration levels of chlorotetain in bacterial supernatants of the *degU* mutants by UHPLC-M. Figure 5d shows that the *degU32* strain produces chlorotetain at higher concentrations than the wild type (B-WT), whereas chlorotetain is not detected in the bacterial supernatant of the *deg146* strain. Altogether, we propose a model where DegU-P promotes bacilysin production by *B. subtilis*, and higher or lower levels of DegU phosphorylation result in increased or reduced bacilysin production, respectively.

### Cell-to-cell attachment indirectly triggers motility

Regarding the factors involved in the *S. epidermidis* triggered *B. subtilis* motility, we investigated whether the intracellular or extracellular content of *S. epidermidis* cells promote the *B. subtilis* motility. To this end, we evaluated the intracellular and extracellular content of *S. epidermidis* cells in a disc assay (Fig. S7) and observed that the extracellular content triggers the *B. subtilis* motility.

**Fig. 6:**
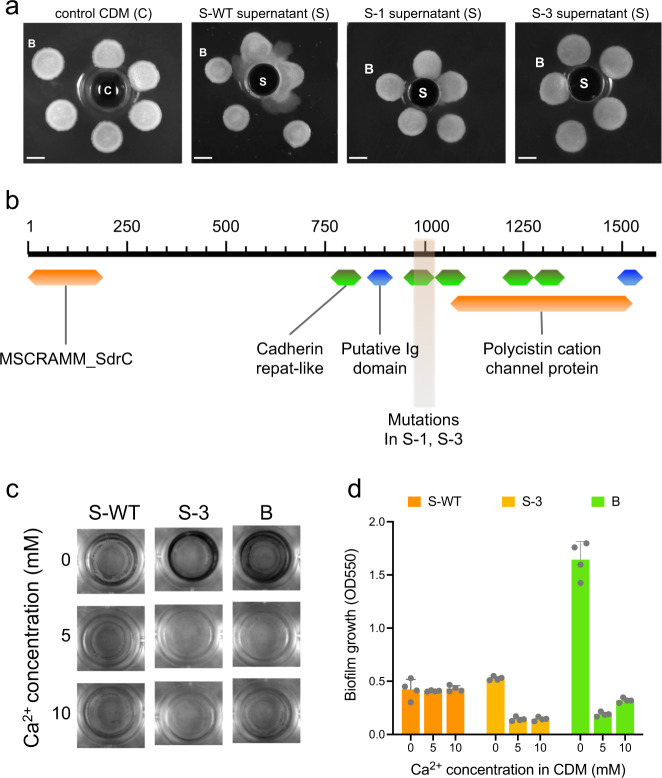
Effects of mutations in the *S. epidermidis* biofilm-associated protein (Bap). **a** Motility assay of *B. subtilis* (B) to the presence of bacterial supernatants of overnight cultures of *S. epidermidis* (S): wild-type (S-WT), and mutants (S-1 and S-3). CDM was used as a control (C; left). Snapshots of colonies on CDM-agar plates incubated at 37 °C for 24 h. Scale bar, 1 mm. **b** Identification of protein domains in the biofilm-associated protein Bap. Domain identification was performed with the InterPro^71^ and NCBI-CDD^72^ databases: MSCRAMM is the acronym for “microbial surface components recognizing adhesive matrix molecules”, which are adhesin proteins that mediate their attachment to surfaces^73^. Cadherin repeat-like motifs are putative Ca^2+^ binding domains^74^. Putative Ig domains are repeats found in several haemagglutinins and other cell surface proteins^75^. Polycystin cation channel protein is a putative Ca^2+^-permeable nonselective cation channel^76^. Location of the mutations present in *bap* of *S. epidermidis* S-1 and S-3 are shown. **c** Qualitative comparison of bacterial cell attachment to polystyrene in a micro-titer plate. The strains *B. subtilis* (B), and *S. epidermidis* wild type (S-WT) and S-3 strains were incubated in CDM supplemented with varying concentrations of Ca^2+^ (0, 5, and 10 mM), at 37 °C for 24 h. The experiment was performed in quadruplicates, and representative images are shown. **d**, Quantitative measurements of bacterial cell attachment (*x-*axis indicates biofilm growth) to polystyrene in a micro-titer plate. The strains *B. subtilis* (B), and *S. epidermidis* wild type (S-WT) and S-3 strains were incubated in CDM supplemented with varying concentrations of Ca^2+^ (0, 5, and 10 mM), at 37 °C for 24 h. Data are presented as mean ± S.D. Error bars represent standard deviation (SD) of the mean values of four experiments.

As mentioned above, *S. epidermidis* S-1 and S-3 are not only resistant to chlorotetain, but also do not promote motility by *B. subtilis*. Therefore, we tested whether overnight *S. epidermidis* supernatants (S-WT, S-1, and S-3) are able to promote motility by *B. subtilis*. To this end, *B. subtilis* cells were spotted in a spiral configuration to show that colonies in the proximity to the supernatant are able to show the typical migratory response. Figure 6a shows that *B. subtilis* colonies (indicated with a B letter) exclusively develop motile cells towards the *S. epidermidis* S-WT supernatant located in the center of the agar plate (indicated with the letter S), compared to the control (CDM indicated with the letter C) and the S-1 or S-3 supernatant. A very low level of *B. subtilis* motility, at the closest distance to the supernatant, is slightly observed towards the S-1 supernatant. Figure 3c shows that the mutations in the biofilm-associated protein are located in a narrow range of residues of the protein sequence (981–1013). Thus, we analyze the Bap protein sequence in order to identify protein domains and gain an insight into the consequences of the mutations on the phenotype of the S-1 and S-3 strains. The sequence analysis reveals that the mutations target a cadherin repeat-like domain (Fig. 6b). Bap proteins are involved in attachment of cells to surfaces and intercellular adhesion. Previous studies show that Bap can sense the bacterial environment through Ca^2+^ concentration changes and trigger the cell-to-cell attachment or biofilm formation^47^. Indeed, cadherin repeat-like motifs are potential Ca^2+^ binding motifs^48^.

Since Bap proteins are responsible for cell-to-cell attachment, and potentially contribute to biofilm formation, we evaluated the attachment of cells of *S. epidermidis* wild type and S-3 strains to polystyrene plates in CDM supplemented with Ca^2+^. We performed a biofilm assay based on crystal violate (CV) in a qualitative (Fig. 6c) and quantitative (Fig. 6d) way, and used *B. subtilis* to compare the biofilm potential of the *S. epidermidis* strains. Figure 6c, d show that cells of *S. epidermidis* S-3 are affected by the presence of Ca^2+^, compared to the wild-type cells (S-WT), which show the same attachment properties when Ca^2+^ is present. Interestingly, the *B. subtilis* biofilm is strongly affected by the presence of Ca^2+^. The effects of Ca^2+^ on colony biofilms of *B. subtilis* have been previously studied, colony expansion is observed at low concentrations of calcium ions and a mechanism where Ca^2+^ modifies the amphiphilic properties of surfactin is proposed^49^.

Furthermore, in a preliminary study between severa*l B. subtilis* and *S. epidermidis* strains, we observed that *B. subtilis* does not develop motility towards a *S. epidermidis* Bap negative strain (Fig. S8). To further confirm this observation, we collected a bacterial supernatant of the *S. epidermidis* Bap negative strain and tested the *B. subtilis* motility as described above. Fig. S9 shows that only the *S. epidermidis* S-WT supernatant (Bap positive) triggers the *B. subtilis* motility. This result further supports a model where the Bap proteins are responsible of promoting *B. subtilis* motility.

Since EDTA is a calcium-chelating agent, we attempted to evaluate the interaction between *B. subtilis* and *S. epidermidis* in CDM-agar plates supplemented with EDTA. To this end, we used CDM containing 2.5 and 0.25 mM EDTA. Fig. S10 shows that EDTA strongly affects the *B. subtilis* growth. *B. subtilis* is unable to grow in the presence of EDTA 2.5 mM, and even at lower EDTA concentration (0.25 mM), where it is able to grow, it is unable to produce chlorotetain and motility. In addition, Fig. S10b shows that the *S. epidermidis* WT colony expansion is higher than the S-3 mutant in the presence of EDTA 0.25 mM. Since the S-3 strain has several mutations in the calcium-binding domain of the Bap protein, we speculate that the phenotype of lower colony expansion is related to the Bap protein and its role in cell-to-cell attachment. A deep biochemical analysis of the Bap protein in the S-1 and S-3 strains to investigate the molecular mechanism involved in the calcium binding or sensing should be undertaken to further corroborate the mechanism.

Together, our data suggest that the mutations in the Bap protein of *S. epidermidis* (S-1 and S-3 strains) potentially affect the cell-to-cell attachment and cell aggregation via Ca^2+^ sensing (see Fig. S4). Since *S. epidermidis* mutants are unable to promote motility in *B. subtilis*, and low concentrations of Ca^2+^ trigger colony expansion in *B. subtilis*^49^, we propose that the *B. subtilis* sliding occurs in response to the low availability of Ca^2+^ ions, which are sequestered by Bap proteins in the proximity of the *S. epidermidis* colony.

## Discussion

**Fig. 7:**
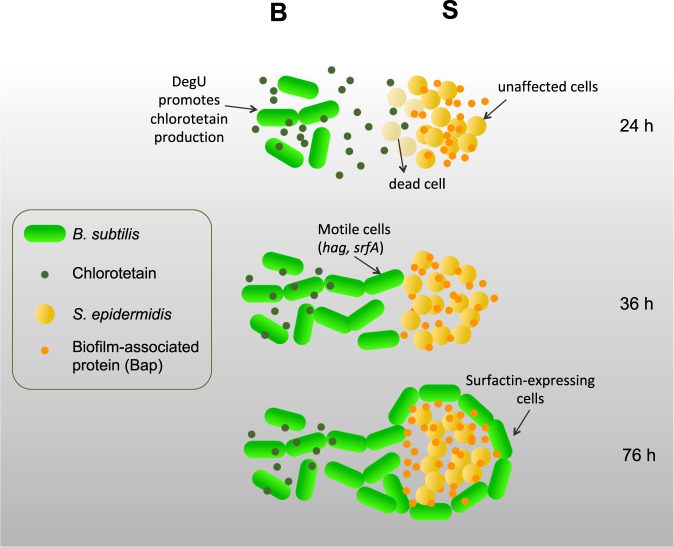
Proposed model of interaction between *S. epidermidis* and *B. subtilis*. Bacterial colonies are able to interact at a close proximity. *B. subtilis* cells (rod-shaped bacteria in green) attack the neighbor colony of *S. epidermidis* (spherical-shaped bacteria in yellow) by producing chlorotetain (indicated with small dark green dots) during the first 24 h of incubation. *S. epidermidis* cells located in the diffusion zone of chlorotetain take the chlorotetain molecules up, and the release of L-anticapsin by chlorotetain hydrolyzation results in bacterial death^41^. However, cells in the periphery of the *S. epidermidis* colony remain unaffected, are able to develop the characteristic cell-to-cell attachment via biofilm-associated proteins (Bap; indicated with small orange dots). The Bap proteins require Ca^2+^ ions to acquire a proper folding and become functional, these proteins also mediate cell attachment to surfaces^47,77^. After 36 to 76 h incubation, the depletion of Ca^2+^ in the surroundings of the *S. epidermidis* colony triggers *B. subtilis* motility. A subpopulation of *B. subtilis* cells emerges, and utilizes sliding motility to reach the *S. epidermidis* colony. The *B. subtilis* engulf the remaining *S. epidermidis* colony, where the presence of Bap might facilitate the *B. subtilis* motility. Since high cell densities of *S. epidermidis* are resistant to chlorotetain, we speculate that the initial unaffected cells reach high cell densities and form biofilms structures, and thus, these cells tolerate the chlorotetain concentrations once the engulfment occurs.

Bacteria commonly live in densely populated environments, where competition for nutrients and space is crucial^10^. The skin is one of these dense environments, where bacterial interactions occur to maintain a balance, and thus determine the composition of our skin microbiota^50^. We use *B. subtilis* and *S. epidermidis* to study whether these bacteria are able to establish a type of specific bacterial interaction. *B. subtilis* is recognized as an important protective bacterium of our skin^16^ and *S. epidermidis* is the most abundant member of the skin microbiota^51^. Our study reveals an interaction between these bacteria based on chlorotetain production and development of *B. subtilis* motile cells. Figure 7 shows a proposed model of this interaction, where the first response from *B. subtilis* colonies (24 h of incubation) is characterized by chlorotetain production, which results in partial degradation of the *S. epidermidis* colony. Previous studies suggest that bacilysin has other cellular functions beyond its antimicrobial activity, for instance bacilysin negative strains are defective in sporulation or germination/outgrowth^52^. In addition, bacilysin is highly produced during starvation stress^30^. Thus, *B. subtilis* might benefit of bacilysin (or chlorotetain) production, when growing in poor-nutrient niches, in two ways: regulation of cellular processes and killing competitors. These observations might explain why to utilize a very simple (dipeptide) antimicrobial instead of others to conquer a nutrient-limited environment. The second response involves an emerging *B. subtilis* subpopulation with sliding motility. Previous studies have demonstrated the capacity of *B. subtilis* to engulf other bacterial species^31,53,54^. We propose that in our study, this motility occurs as a response to the low Ca^2+^ levels in the environment due to the Ca^2+^ binding to the biofilm-associated protein Bap within the *S. epidermidis* colony and its surroundings. We propose that this spreading property by *B. subtilis* is a niche conquering strategy to expand its bacterial colony and limit the growth of other species.

Bacterial interactions are shaped by several factors, for instance by the cell density and the proximity between cells^55^. In our study, the interactions between *B. subtilis* and *S. epidermidis* colonies are indeed affected by the initial cell density. For instance, we observed that the lower the *S. epidermidis* initial cell densities, the higher the colony degradation (Fig. S11). This effect can be explained by the fact that at low *S. epidermidis* cell densities, chlorotetain is quickly produced by *B. subtilis* (present at higher cell densities), then it accumulates in the growth medium and immediately kills the low number of *S. epidermidis* cells.

The avoidance of conflicts and fights is a common strategy to survive between animals^56–58^. Here, we uncover an interaction between two microorganisms that resembles examples of the conflicts observed between higher organisms, for instance when an animal backs down if faced with another animal in competition. In this case study, our data suggest that *S. epidermidis* is actually playing a role in the interaction with *B. subtilis* by limiting the incoming attacks, e.g., it develops resistance to chlorotetain and mutations in the *bap* gene arises, which results in abolishment of the *B. subtilis* invasion of its colony. This strategy results in an effective way to allow *S. epidermidis* to survive. We observed that *S. epidermidis* resistant colonies show lower growth rate and reach lower cell densities compared to the wild type (Fig. S12). Thus, the resulting genetic mutations on the *dtpT* and *bap* genes lower the *S. epidermidis* fitness probably because of their impact on essential processes such as the nutrient uptake (via DtpT), and cell-to-cell adhesion (via Bap). Furthermore, as mentioned above, cell density also plays an important role in this interaction. Thus, the unaffected *S. epidermidis* cells (initially at a far distance from the *B. subtilis* colony), might not develop chlorotetain resistance, but are able to quickly grow and reach a high cell density, becoming a bacterial population able to tolerate high chlorotetain concentrations.

This study sheds light on how microbial communities define their territory in certain microenvironments of our skin, where many other bacteria live. Notably, *B. subtilis* is able to produce a plethora of antimicrobial compounds, which show activity against diverse bacteria and fungi^14,59^. For instance *B. subtilis* produces bacillomycin, an antibiotic with antifungal activity against all the important dermatophytes and systemic fungi^60^. Likewise *S. epidermidis* is able to produce peptides to inhibit *Staphylococcus aureus* and *Streptococcus pyogenes*^2^.

The skin microbiota is composed of many other different species of bacteria^2,3^. Thus, it is interesting to speculate about different interaction scenarios between these bacteria. Our study shows how bacteria respond locally to the secreted products of others, for instance the *S. epidermidis* resistance response when inhibitory molecules are in the environment. In addition, species of bacteria are known to colonize specific microenvironments of our skin, based on different factors such as pH, nutrients, or humidity^61^. Accordingly, specific bacterial competition strategies appear to be an important factor that defines which bacteria colonize a specific microenvironment of our skin.

## Methods

### Bacterial strains and growth conditions

All the bacterial strains used for this study are listed in the Supplementary Table 1. Experiments were performed with *Bacillus subtilis* 168, and *Staphylococcus epidermidis* JH (wild type). *Staphylococcus epidermidis* JH was isolated from human skin in Groningen, The Netherlands, by using an inoculation loop, passing it onto the surface of a human arm, and then streaking an M17-agar plate. A colony with beige color was obtained after 24 h incubation at 37 °C. The colony was again streaked out on an M17-agar plate to ensure the isolation of a single bacterium. Initially, the sequence analysis of 16S rRNA gene was performed using the BLAST program to identify this bacterium as *Staphylococcus epidermidis*^62^. Confirmation of the taxonomic assignment was performed by genomic DNA sequencing (see Genome sequencing).

*B. subtilis*, *S. epidermidis, C. acnes, S. thermophilus* cells were grown at 37 °C in chemically defined medium (CDM)^63^, supplemented with glucose (Sigma-Aldrich) 0.5% (w/v). CDM contained 49.6 mM NaCl, 20.1 mM Na_2_HPO_4_, 20.2 mM KH_2_PO_4_, 9.7 μM (±)-α-lipoic acid, 2.10 μM D-pantothenic acid, 8.12 μM nicotinic acid, 0.41 μM biotin, 4.91 μM pyridoxal hydrochloride, 4.86 μM pyridoxine hydrochloride, 2.96 μM thiamine hydrochloride, 0.24 μM (NH_4_)_6_Mo_7_O_24_, CaCl_2_ 20.4 μM, 1.07 μM CoSO_4_, 1.20 μM CuSO_4_, 1.04 μM ZnSO_4_, 20.12 μM FeCl_3_, 1.46 mM L-alanine, 1.40 mM L-arginine, 0.61 mM L-asparagine, 1.03 mM L-aspartic acid, 0.35 mM L-cysteine, 0.66 mM L-glutamic acid, 0.66 mM L-glutamine, 0.39 mM glycine, 0.16 mM L-histidine, 0.63 mM L-isoleucine, 0.89 mM L-leucine, 1.02 mM L-lysine, 0.27 mM L-methionine, 0.39 mM L-phenylalanine, 3.58 mM L-proline, 1.64 mM L-serine, 0.57 mM L-threonine, 0.18 mM L-tryptophan, 2.76 mM L-tyrosine and 0.73 mM L-valine. GM17-agar or CDM-agar plates were prepared by adding agar 1.5% (w/v) and glucose to M17 or CDM, respectively. When necessary, culture media was supplemented with erythromycin 5 µg mL^−1^, chloramphenicol 5 µg mL^−1^, kanamycin 10 µg mL^−1^ or spectinomycin 100 µg mL^−1^.

*Lactococcus lactis* cells were grown at 30 °C in M17 broth (Difco^TM^ BD, NJ, USA) or in CDM, supplemented with glucose (Sigma-Aldrich) 0.5% (w/v).

For microscopy experiments or plate-reader assays, bacterial strains were grown in CDM with glucose 0.5% (w/v) and collected by centrifugation from exponential growth cultures (optical density of 0.4 at 600 nm) and washed three times with phosphate-buffered saline (PBS) solution (pH 7.2) containing: 15.44 µM KH_2_PO_4_, 1.55 mM NaCl and 27.09 µM Na_2_HPO_4_.

### DNA techniques and oligonucleotides

DNA amplifications by PCR were performed using a PCR mix containing Phusion HF Buffer (Thermo Fisher Scientific Inc., MA, USA), 2.5 mM dNTPs mix, Phusion HF DNA polymerase (Thermo Fisher Scientific Inc., MA, USA), primers (0.5 μM each), and 50 ng of *S. epidermidis* JH chromosomal DNA as template. Oligonucleotides (Supplementary Table 2) were purchased from Biolegio (Nijmegen, The Netherlands). PCRs were performed in an Eppendorf thermal cycler (Eppendorf, Hamburg, Germany). The 16 s rRNA was amplified by 35 cycles of denaturation (98 °C for 30 s), annealing (5 °C or more, lower than *T*_m_ for 30 s), and extension (70 °C for 1 min per 1 Kbp). Amplifications were confirmed by 1% (w/v) agarose gel electrophoresis method. PCR products were isolated and cleaned-up with a high pure plasmid isolation kit (Roche Applied Science, Mannheim, Germany), according to the protocol of the manufacturer. DNA sequences were obtained by sequencing of the PCR fragments in the genomic region of interest (Macrogen Europe, Amsterdam, The Netherlands).

### Interaction assay

Bacterial interaction assays were performed on CDM-agar plates. *B. subtilis* and *S. epidermidis* cells were grown overnight in CDM and washed three times with PBS. The optical density at 600 nm of each bacterial growth was adjusted to 0.4. A volume of 5 μL of each bacterial suspension was spotted on CDM-agar plates at different distances and incubated at 37 °C.

To test swarming, swimming and sliding motility in *B. subtilis*, CDM-agar plates were prepared with different agar concentrations: 0.8, 1, 1.5 and 2% (w/v).

### Microscopy observations

Interaction between colonies of *B. subtilis* and *S. epidermidis lactis* was detected using an Olympus MVX20 macro zoom fluorescence microscope equipped with a PreciseExcite light-emitting diode (LED) fluorescence illumination (470 nm), GFP filter set (excitation 460/480 nm and emission 495/540 nm). Bright-field and fluorescent images were acquired with an Olympus XM10 monochrome camera (Olympus Co., Tokyo, Japan).

### DNA sequencing

A single colony *S. epidermidis* JH (wild type strain) growing on a CDM-agar plate was selected and grown as standing culture in 5 mL of CDM broth, supplemented with 0.5% (w/v) glucose, and incubated overnight at 37 °C. Cells from the bacterial culture were collected by centrifugation at 6000×*g* for 3 min in a Microfuge 16 centrifuge (Beckman Coulter, Woerden, The Netherlands). Genomic DNA was isolated with a GenElute bacterial genome DNA kit (Sigma-Aldrich, Munich, Germany) according to the manufacturer’s instructions. The resulting genomic DNA was used as template to perform PCRs. The 16S rRNA gene was amplified by colony PCR using the oligonucleotides 27 F and 1492 R (see Supplementary Table 2). The DNA sequences were analyzed with BLAST program^62^.

In addition, the genomic DNA of the *S. epidermidis* mutant strains (S-1 and S-3) was isolated as described above. To this end, all the staphylococci genomes (wild type, S-1 and S-3) were paired-end sequenced at the Beijing Genomics Institute (BGI, Copenhagen N, Denmark) on a BGISEQ-500 platform. A total of 5 million paired-end reads (150 bp) were generated. FastQC version 0.11.5^64^ was used to examine the quality of the reads. Taxonomic assignment of reads was performed with Kraken 2.0.7^65^. The Rapid Annotations using Subsystems Technology (RAST) server^66^ was used to annotate the genomes. Identification of mutations was performed with Breseq^67^, using the *S. epidermidis* JH data as a reference sequence (JAAUOD000000000).

### Antimicrobial assay

Each *B. subtilis* strain was inoculated in 100 mL of CDM and grown at 37 °C. Cells were harvested by centrifugation at 6000×*g* for 15 min. The supernatants were transferred into a clean tube, filtered through nitrocellulose Whatman filters (0.45 and 0.2 µm) and stored at 4 °C for subsequent analysis. Antimicrobial activity of 150 µL of the bacterial supernatants was assessed on CDM-agar plates, using *S. epidermidis* wild-type strain as indicator strain.

### Chlorotetain identification

UHPLC-M analysis was performed using a Dionex Ultimate 3000 UHPLC chromatographic system combined with a Q Exactive mass spectrometer (both from Thermo Fisher Scientific) fitted with a heated electrospray source operated in the positive ion mode. UHPLC separation was performed on a Kinetex EVO C18, 2.6 µm, 2.1 mm × 150 mm column (Phenomenex, Maarsen, The Netherlands). The column was kept at 50 ± 0.1 °C during analysis. Mobile phases were A: 100% water, 0.1% formic acid, and B: 100% ACN and 0.1% formic acid. The injection volume for all separations was 10 µL. Chromatographic elution was achieved under gradient conditions with a flow rate of 400 µL/min. Elution started with an isocratic step of 1.0 min at 1% B, followed by a linear gradient from 1 to 95% B (1.0–10.0 min. These conditions were maintained for 2 min before returning to 1% B in 0.03 min and equilibration at start conditions for 3 min. The total runtime was 15 min. The Q Exactive mass spectrometer was operated with a capillary voltage of 3.50 kV. The capillary temperature was set at 275 °C, and auxiliary gas temperature at 350 °C. The sheath gas pressure, auxiliary gas pressure and sweep gas flow rate were set at 50, 10, and one arbitrary units, respectively, with nitrogen gas. Spectra were recorded from 100 to 750 m/z at a resolution of 70000@mz200. Xcalibur 4.1 was used for data processing.

### Motility assay

*B. subtilis* motility was assessed on CDM-agar plates. One fifty microliter of filtered *S. epidermidis* supernatants were located in the center of the CDM-agar plates, and 5 µL of *B. subtilis* was spotted on the plates around the *S. epidermidis* supernatant in a spiral configuration to evaluate motility at different diffusion distances. A plate with 150 µL of CDM instead of bacterial supernatant was used as a control. CDM-agar plates were incubated 24 h at 37 °C.

### Disc assay

Sterile 5.5 mm diameter filter paper discs were placed on CDM-agar plates. Two discs were used per plate. Twenty microliters of the bacterial supernatant or control medium (CDM) were placed on the center of the discs, and the plates were dried for 5 min by keeping them open in a laminar flow cabinet. Five microliters of an overnight culture of the *B. subtilis strain* was then inoculated at different distances to the edge of the discs. The plates were incubated at 37 °C for 24 h. After the incubation period, snapshots were taken with an imaging system ChemiDoc XRS (Bio-Rad).

### Intracellular and extracelular contents

Each *S. epidermidis* strain (S-WT, S-1, S-3) was inoculated in 20 mL of CDM and grown overnight at 37 °C. Cells were harvested by centrifugation at 6000×*g* for 15 min. The supernatants were transferred into a clean tube, filtered through nitrocellulose Whatman filters (0.45 and 0.2 µm) and stored at 4 °C for subsequent analysis. Motility activity of *B. subtilis* was tested with 150 µL of the bacterial supernatants (extracellular) on CDM-agar plates. For intracellular content, the bacterial pellet was resuspended in CDM (cell suspension) and sonicated on ice for 10 min using a VCX 130 Sonicator with cycle 10 s ON and 10 s OFF. Cell debris was removed by centrifugation at 6000×*g* for 15 min at 4 °C. The supernatant was collected and filtered through a 0.45 μm filter.

### Biofilm assays

We employed a biofilm formation method using the dye crystal violet (CV)^68^. Bacterial strains were grown overnight in CDM supplemented with different calcium concentrations (1, 5, and 10 mM). Grown was performed in 100 µL per well in a 96-well plate. We used four replicates per treatment. The plate was incubated 26 h at 37 °C. After incubation, bacterial cultures were removed from the wells and rinsed two times with deionized water to remove unattached cells. Next, 125 μL of a 0.1% solution of crystal violet in water to each well of the micro-titer plate was added. The micro-titer plate was incubated at room temperature for 15 min. Then, it was rinsed three times with water, and dried by incubation at 37 °C for 2 h. For qualitative assays, the wells were photographed. Qualitative measurements were performed as follows. One twenty five microliter of 30% acetic acid in water to each well were added to solubilize the CV. The micro-titer plate was incubated at room temperature for 15 min. One twenty five microliter of the solubilized CV was transferred to a new flat-bottomed micro-titer plate. Absorbance measurements were performed in a plate reader at 550 nm using 30% acetic acid in water as the blank.

### Plate-reader assays

Cultures of *S. epidermidis* and *B. subtilis* were grown and prepared as described above. For growth measurements, cells were diluted 1:20 in CDM. The growth was recorded in 0.2 mL cultures in 96-well micro-titer plates and monitored by using a micro-titer plate reader VarioSkan (Thermo Fisher Scientific Inc., MA, USA). Growth was recorded with measurements of the optical density at 600 nm (OD_600_) every 10 min for 24 h. Values were corrected for the background value of the corresponding medium used for growth.

### Flow cytometry

*B. subtilis* strains were grown for interaction assays with *S. epidermidis* in CDM as described above. Biofilms were scraped from the CDM-agar surface and resuspended in 800 µL PBS. The biofilms were disrupted by repetitive passes through a 23 G needle as reported in a previous study^69^. Cells were subjected to a mild sonication to obtain a preparation of single cells as described^70^. A threshold for the FSC and SCC parameters was set (200 in both) in the FACS Canto flow cytometer (BD Biosciences, CA, USA) to remove all the events that do not correspond to cells. The GFP-signal at all the measured cells was recorded in 30,000 events and used for downstream analysis (named ungated events in the corresponding figures). GFP-signal measurements were obtained with a FACS Canto flow cytometer (BD Biosciences, CA, USA) using a 488 nm argon laser. Raw data was collected using the FACSDiva Software 5.0.3 (BD Biosciences). And the FlowJo software was used for data analysis (https://www.flowjo.com/).

### Statistics and reproducibility

Statistical analyses were performed using Prism 6.01 (GraphPad software https://www.graphpad.com/). All experiments were repeated independently at least three times. All micrographs, including small insets, show representative images from three independent replicate experiments.

### Bioinformatics

Alignments and sequences identities were determined by using BLAST^62^ and RAST^66^, using the full-length protein or DNA sequences. Protein domains were identified with the InterPro and NCDI-CDC databases. Identification of mutations was performed with breseq version 0.32.1^67^.

### Reporting summary

Further information on research design is available in the Nature Research Reporting Summary linked to this article.

## Supplementary material

Supplementary Information

Reporting Summary

## Data Availability

The *S. epidermidis* JH, S-1 and S-3 genomes were submitted to NCBI and are publicly available in the following accession codes: JAAUOD000000000 (JH; WT), JABTXG000000000 (S-1) and JABTXF000000000 (S-3). Data supporting the findings of this work are available within the paper and its Supplementary Information files. All other data are available from the corresponding author on request.
