# Supplementary material for: Impact of spatial proximity on territoriality among human skin bacteria
Source: NPJ Biofilms Microbiomes. 2020 Aug 6;6:30. doi: 10.1038/s41522-020-00140-0 (PMC7413532; doi:10.1038/s41522-020-00140-0)
Supplement: Supplementary file 1 — Supplementary Information [file 41522_2020_140_MOESM1_ESM.pdf]

Supplementary Information

Impact of spatial proximity on territoriality among human skin bacteria

Hernandez-Valdes *et al*.

Supplementary Figures

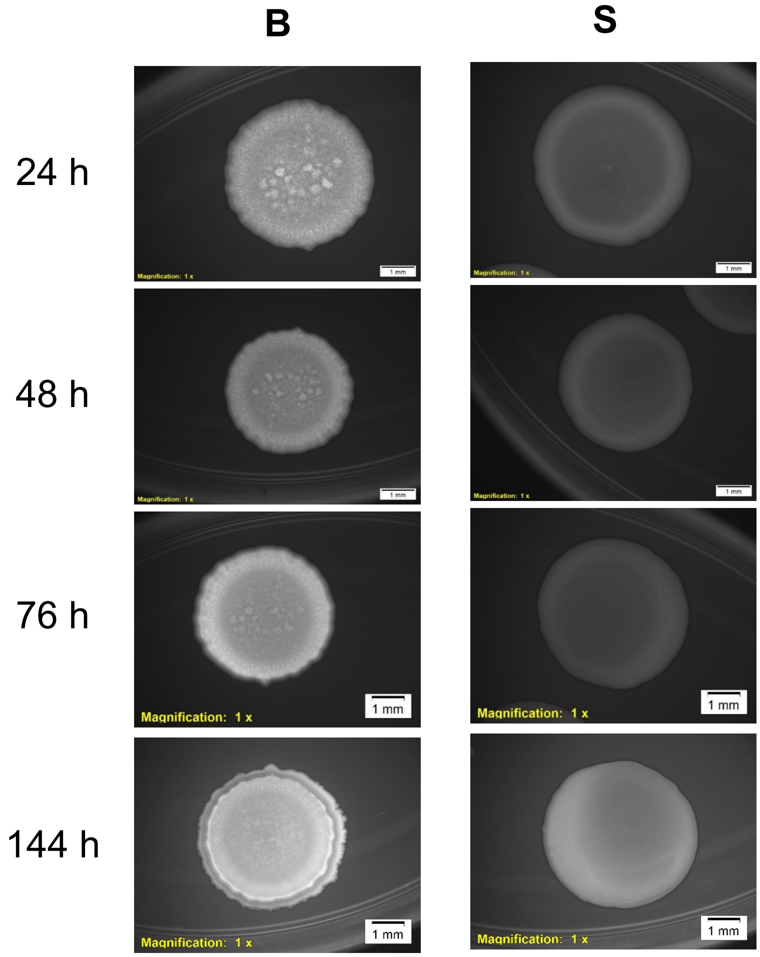

Supplementary Figure 1 | Independent growth of *B. subtilis* and *S. epidermidis* on CDM-agar plates. Bacterial colonies of *B. subtilis* (B) and *S. epidermidis* (S) on independent CDM-agar plates, after different incubation times (24, 48, 72, and 144 h) at 37 °C. Snapshots of fluorescence microscope observations are shown. Scale bar, 1 mm.

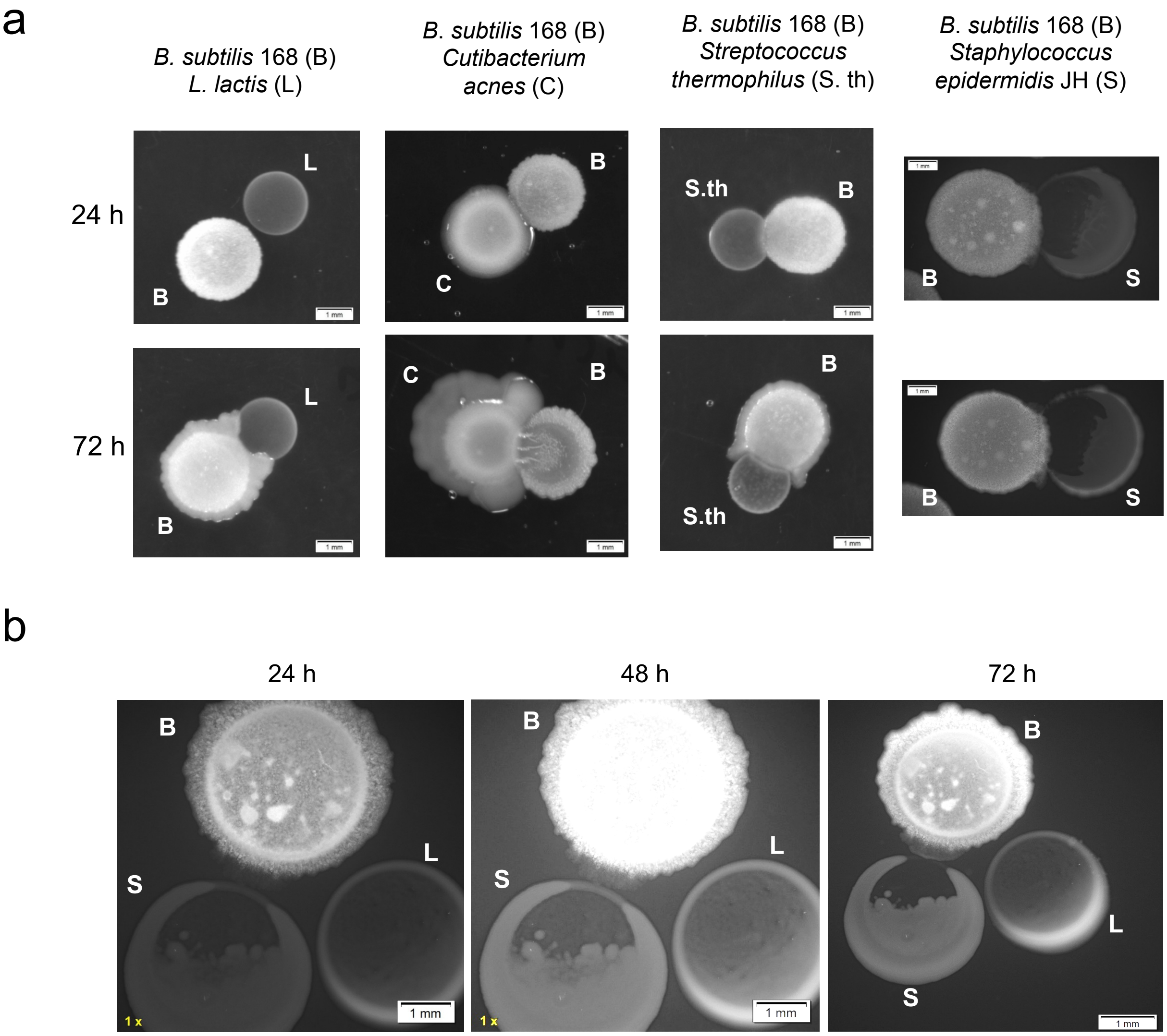

Supplementary Figure 2 | Interaction assays between *B. subtilis* and other bacteria. a, Bacterial colonies of *B. subtilis* (B), *L. lactis* (L), *S. thermophilus* (S.th) and *S. epidermidis* (S) grown on CDM-agar plates, after different incubation times (24 and 72 h) at 30 °C. Scale bar, 1 mm. **b**, Interaction between *B. subtilis* (B), *L. lactis* (L), and *S. epidermidis* (S) grown on a CDM-agar plate, after different incubation times (24, 48 and 72 h) at 30 °C. Snapshots were taken with the imaging system ChemiDoc XRS (Bio-Rad) or from fluorescence microscopy observations. Scale bar, 1 mm.

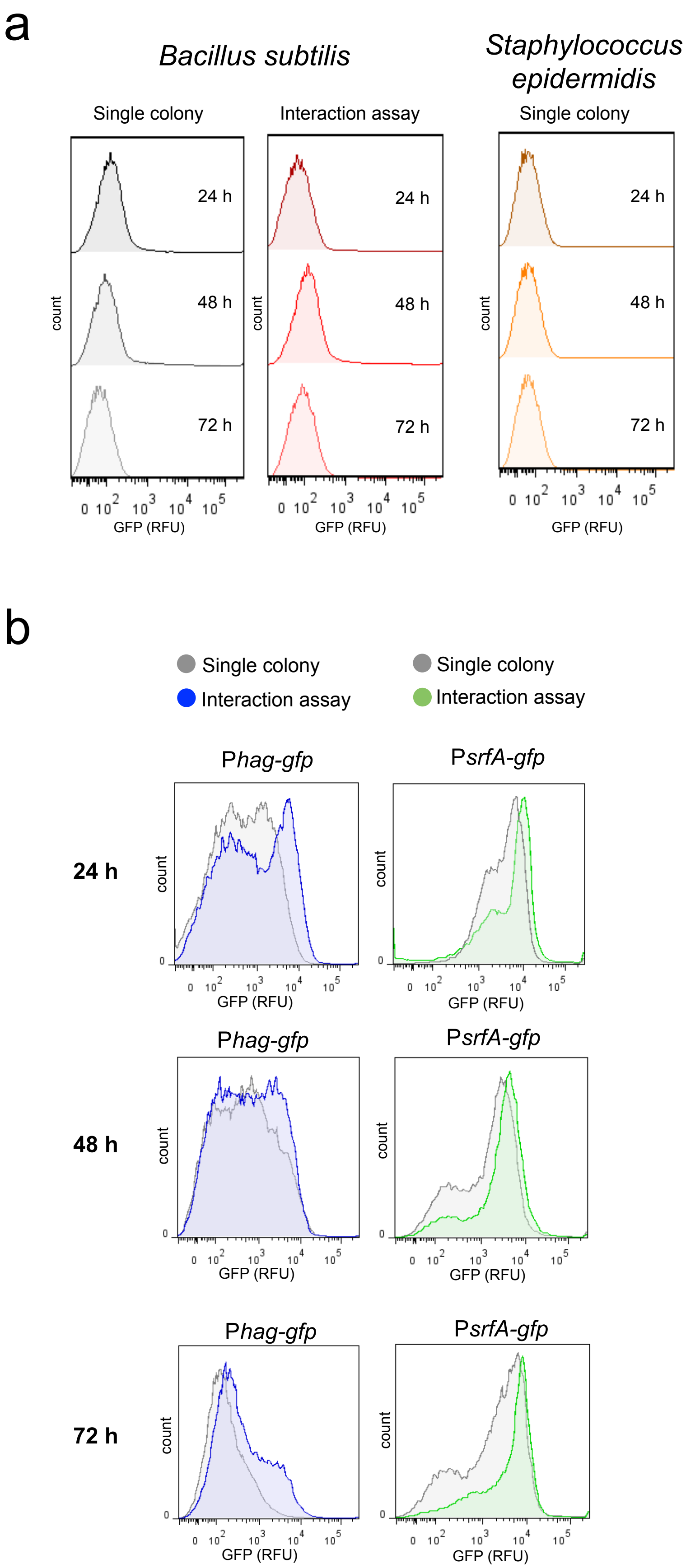
a
Bacillus subtilis
Staphylococcus epidermidis
Single colony
Interaction assay
Single colony
24 h
48 h
72 h
count
GFP (RFU)
b
Single colony
Interaction assay
Single colony
Interaction assay
Phag-gfp
PsrfA-gfp
24 h
48 h
72 h
count
GFP (RFU)

Supplementary Figure 3 | Single-cell GFP measurements in single colonies and interaction assays between *B. subtilis* and other bacteria. a, Background GFP expression of single bacterial colonies of *B. subtilis* (B) and *S. epidermidis* (S) grown on CDM-agar plates, after different incubation times (24, 48 and 72 h) at 37 °C. **b,** Comparison of *hag* and *srfA* expression, by flow cytometry, between single *B. subtilis* colonies (in grey color) and *B. subtilis* cells in and interaction assay with *S. epidermidis* (in blue for *hag* expression, and in green for *srfA* expression), grown on CDM-agar plate, after different incubation times (24, 48 and 72 h) at 37 °C. 30,000 ungated events for each sample are shown.

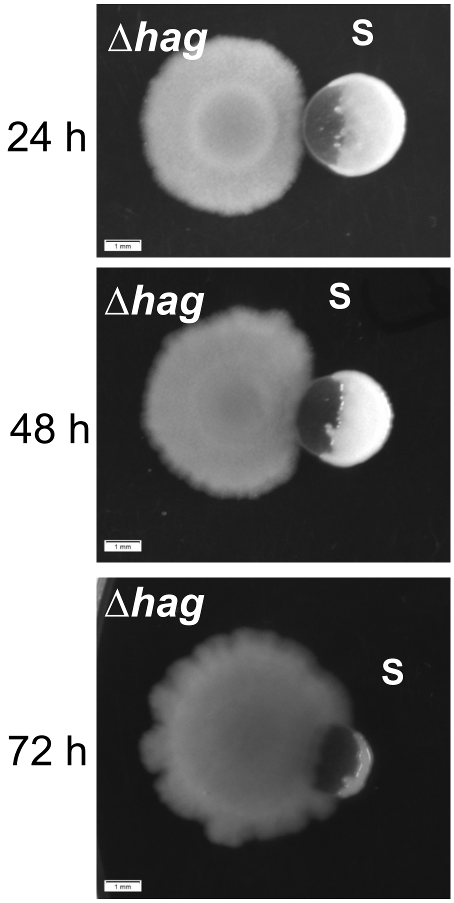

Supplementary Figure 4 | Interaction assay with the *B. subtilis hag* deletion mutant. Interaction assays with bacterial colonies of *B. subtilis* Δ*hag* and *S. epidermidis* (S) grown on a CDM-agar plate, after different incubation times (24, 48 and 72h) at 37 °C. Scale bar, 1 mm. A migratory response is observed after long incubation time (72 h). Snapshots were taken with the imaging system ChemiDoc XRS (Bio-Rad).

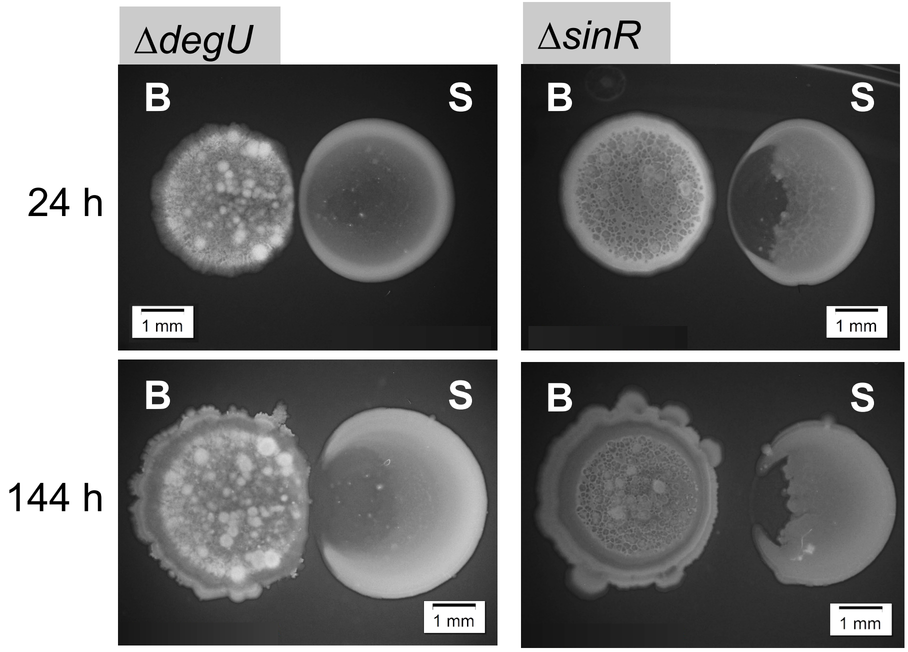

Supplementary Figure 5 | Interaction assay with *B. subtilis sinR* and *degU* mutants. Interaction assays with bacterial colonies of *B. subtilis* (Δ*degU* and Δ*sinR*), and *S. epidermidis* (S) grown on a CDM-agar plate, after different incubation times (24 and 144 h) at 37 °C. A slight growth inhibition is observed after a long incubation time (144 h) in the Δ*degU* assay; this result can be explained by residual bacilysin (chlorotetain) production by other regulators that bind the *bac* operon such as CodY. Snapshots of fluorescence microscope observations are shown. Scale bar, 1 mm.

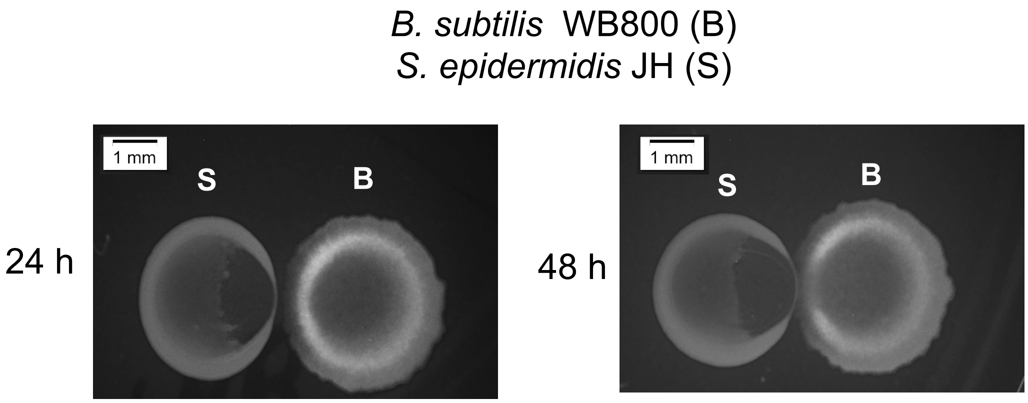

Supplementary Figure 6 | Interaction assay with *B. subtilis* WB800. Interaction assays with bacterial colonies of *B. subtilis* WB800 (*nprE*, *nprB*, *aprE*, *epr*, *bpr, mpr*, *vpr*, *wprA*) and *S. epidermidis* (S) grown on a CDM-agar plate, after different incubation times (24 and 48 h) at 37 °C. Snapshots of fluorescence microscope observations are shown. Scale bar, 1 mm.

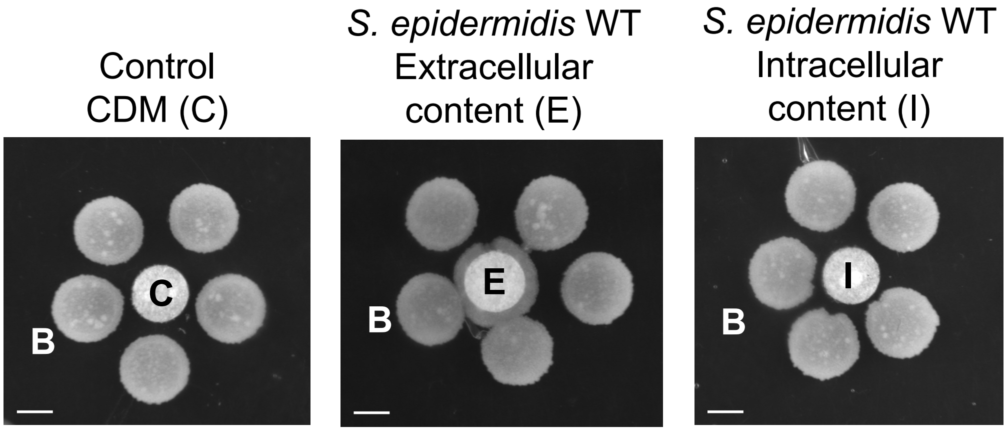

Supplementary Figure 7 | The *B. subtilis* motility is triggered by the extracellular content of *S. epidermidis* cells. Disc assay with intracellular (I) and extracellular (E) content of *S. epidermidis* cells. Twenty microliters of CDM or bacterial content (E and I) were placed in the disc. Five microliters of *B. subtilis* cells (see Methods) were placed at different distance to the edge of the disc to test motility. Snapshots were taken after 24 h incubation at 37 °C with the imaging system ChemiDoc XRS (Bio-Rad). Scale bar, 1 mm.

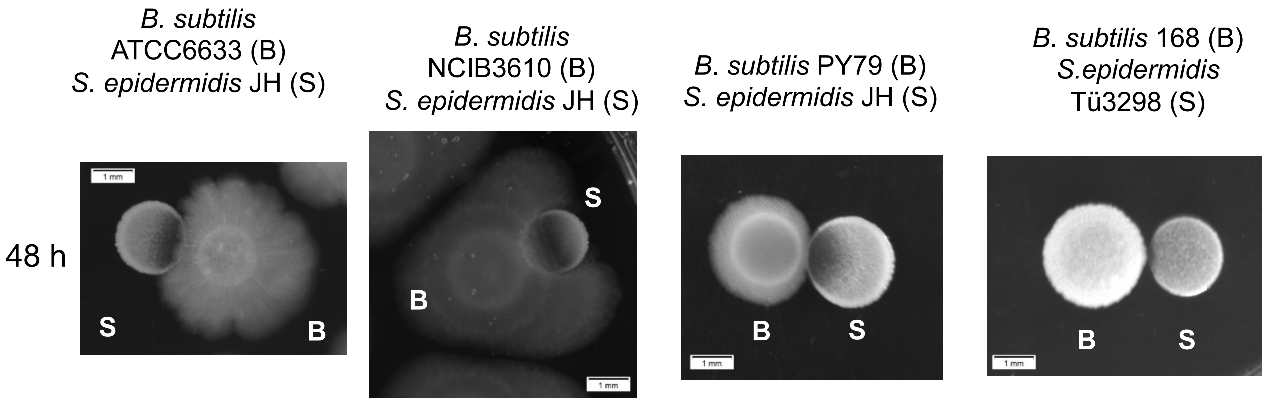

Supplementary Figure 8 | Interaction assays between different *B. subtilis* and *S. epidermidis* strains. Interaction between three different *B. subtilis* strains (ATCC6633, NCIB3610, PY79 and 168) with *S. epidermidis* JH strain. In addition, an interaction between *B. subtilis* 168 and the *S. epidermidis* (Bap negative) Tü3298 strain is shown. Grown on a CDM-agar plate for 48 h at 37 °C. Snapshots were taken with the imaging system ChemiDoc XRS (Bio-Rad) or from fluorescence microscopy observations. Scale bar, 1 mm.

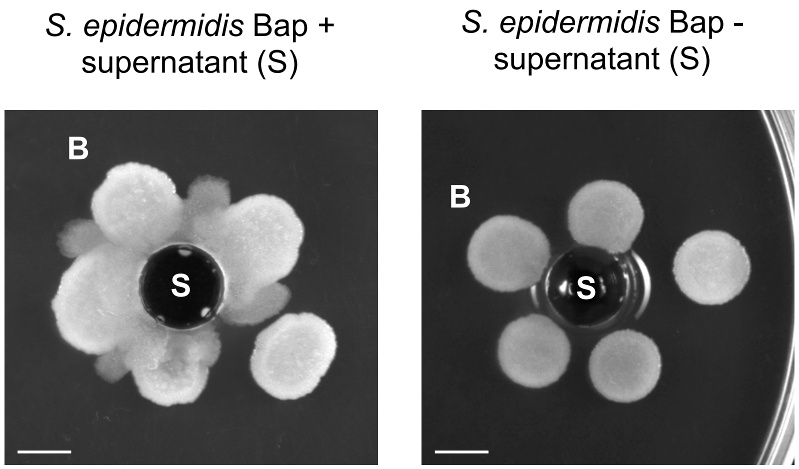

Supplementary Figure 9 | Motility assay using bacterial supernatants of a *S. epidermidis* Bap positive and negative strains. *S. epidermidis* JH (Bap positive) strain and *S. epidermidis* (Bap negative) Tü3298 strain. *B. subtilis* cells were spotted and grown on a CDM-agar plate for 24 h at 37 °C. Snapshots were taken with the imaging system ChemiDoc XRS (Bio-Rad). Scale bar, 1 mm.

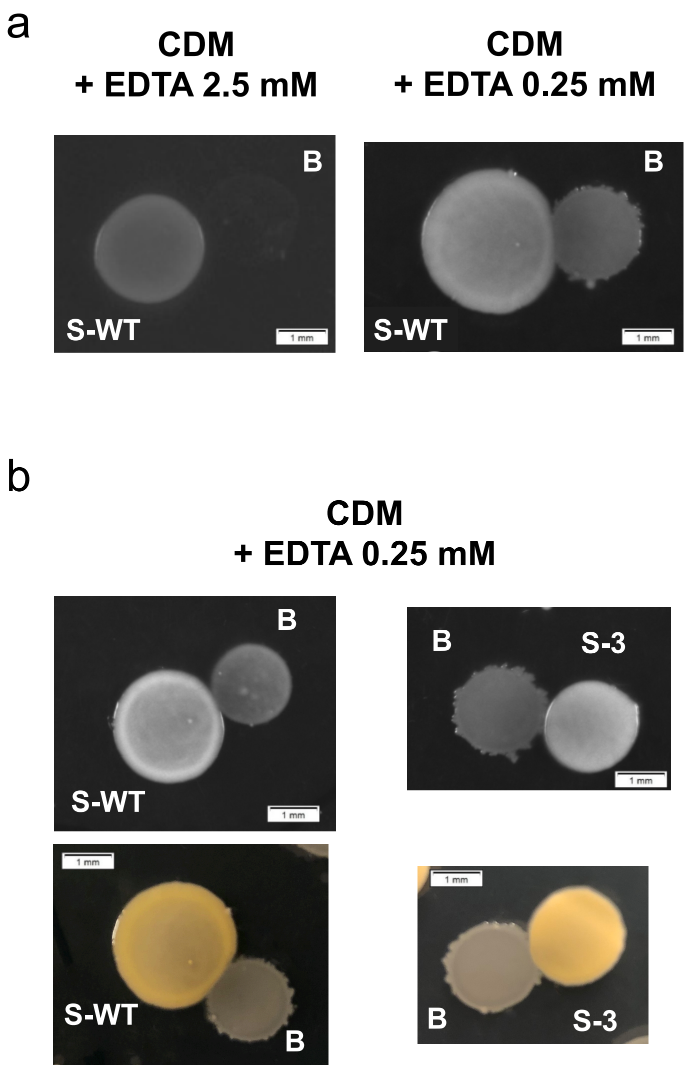

Supplementary Figure 10 | Interaction assays on EDTA-containing CDM-agar plates. **a,** Two EDTA concentrations were tested, *B. subtilis* is unable to grow at 2.5 mM EDTA, in contrast to *S. epidermidis,* which shows good growth in either 2.5 or 2.5 mM. Scale bar, 1 mm. **b,** Comparison between the interactions of *S. epidermidis* S-WT and S-3 with *B. subtilis*. Bacterial cells were spotted and grown on a CDM-agar plate for 24 h at 37 °C. Snapshots were taken with the imaging system ChemiDoc XRS (Bio-Rad) and bright-field microscopy. Scale bar, 1 mm.

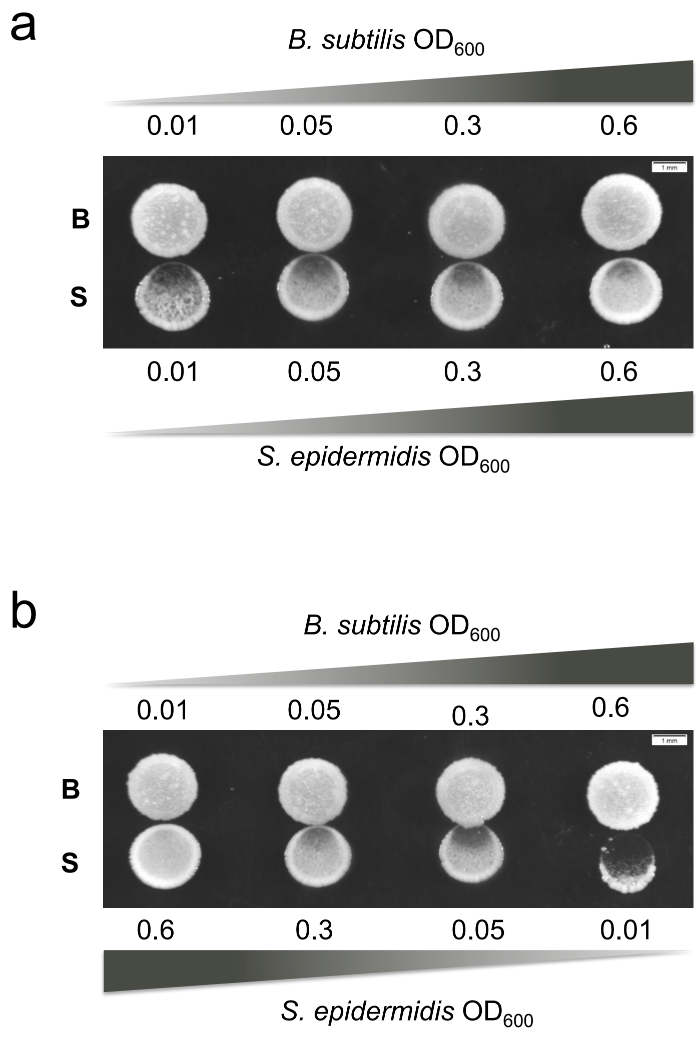

Supplementary Figure 11 | Effect of cell density on chlorotetain production, a and **b,** *B. subtilis* and *S. epidermidis* cells were spotted at low and high cell densities, and their interaction was tested in either similar cell densities (a) or opposite cell densities (b). Optical cell densities at 600 nm values are shown. Bacterial cells were spotted and grown on a CDM-agar plate for 24 h at 37 °C. Snapshots were taken with the imaging system ChemiDoc XRS (Bio-Rad). Scale bar, 1 mm.

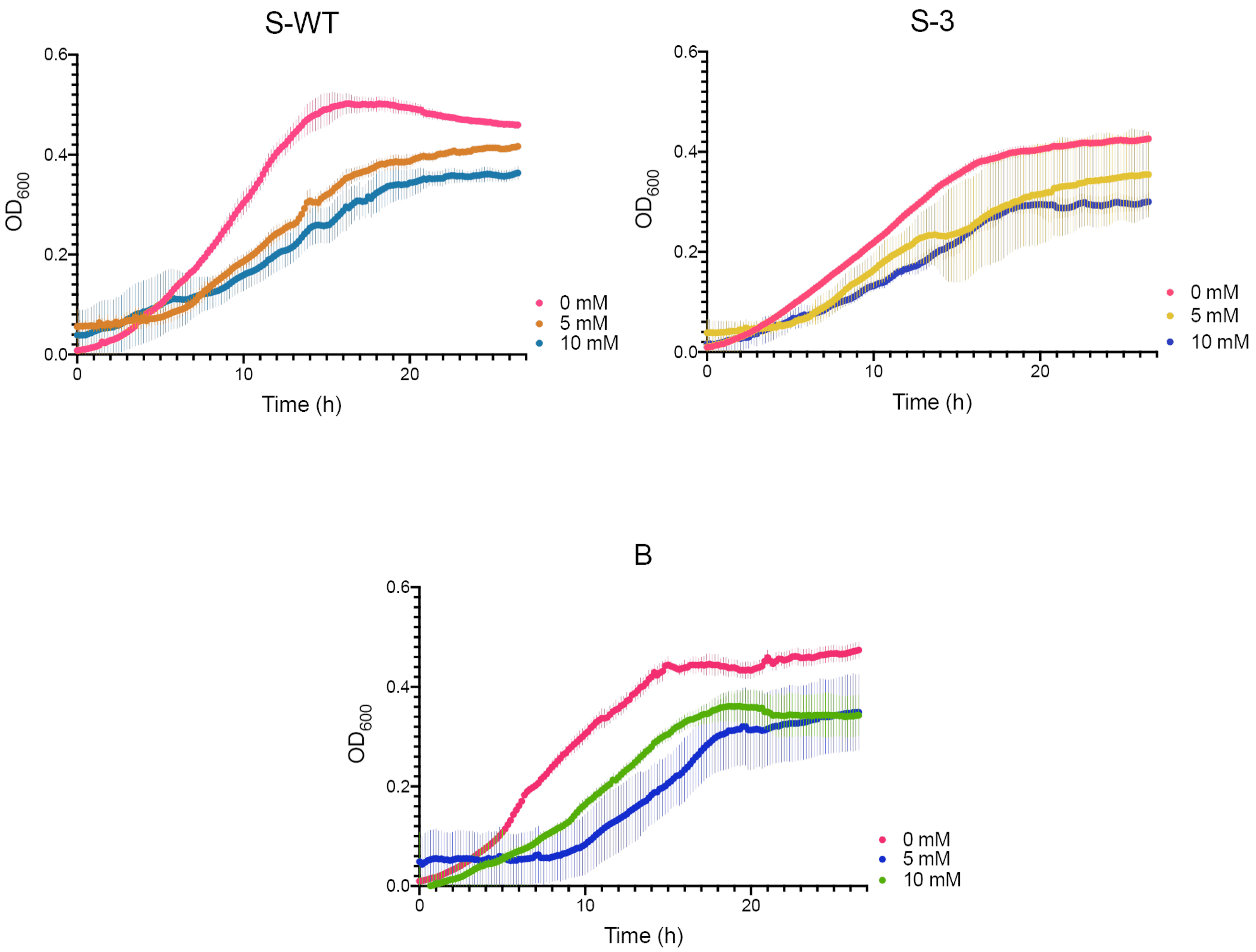

Supplementary Figure 12 | Effect of calcium on bacterial growth. The strains *B. subtilis* (B), and *S. epidermidis* wild type (S-WT) and S-3 strains were incubated in CDM supplemented with varying concentrations of Ca^{2+} (0, 5 and 10 mM), at 37 °C for 24 h. Data are presented as mean ± S.D. Error bars represent standard deviation (SD) of the mean values of four experiments. Note that the S-3 strain shows a lower growth rate and reaches lower cell densities compared to the wild type (S-WT) in CDM (Ca^{2+} 0 mM).

Table S1. Strains used in this study

Strain	Description	Reference
Bacillus subtilis 168	*trpC2*	[1]
Staphylococcus epidermidis JH	Wild-type strain isolated from human skin	This study
Lactococcus lactis MG1363	Opp^+, $DtpT^+$, Dpp^+, Lac^-, Prt^-; *L. lactis* subsp. *cremoris,* plasmid-free NCDO712.	[2]
GFP+ *B. Subtilis* WT	Cm^R, *B. subtilis* 168 strain bearing a P*veg-gfp* construct. Genomic integration by single recombination event in the *amyE* locus. The *veg* gene is a housekeeping gene.	Gift from Imke van Baarle
B. subtilis P*srfA-gfp*	Cm^R*, B. subtilis* 168 strain bearing a P*srfAA-gfp* construct.	[3]
B. subtilis P*hag-gfp*	Cm^R*, B. subtilis* 168 strain bearing a P*hag-gfp* construct.	[3]
B. subtilis P*bslA-gfp*	Cm^R*, B. subtilis* 168 strain bearing a P*bslA-gfp* construct.	[3]
B. subtilis P*bpr-gfp*	Cm^R*, B. subtilis* 168 strain bearing a P*bpr-gfp* construct.	[3]
B. subtilis **Δ***tasA*	Sp^R*, B. subtilis* 168 *tasA::*pBL601	Gift from Marielle van den Esker
B. subtilis **Δ***epsG*	Sp^R*, B. subtilis* 168 *epsG::*pBL601	[4]
B. subtilis **Δ***srfA*	Km^R*, B. subtilis* 168 *srfAA::aphA3*	Gift from Emmo Koetje
B. subtilis **Δ***sinR*	Sp^R*, B. subtilis* 168 *sinR::*pBL601	[5]
B. subtilis **Δ***hag*	Km^R*, B. subtilis* 168 *hag::aphA3*	[6]
B. subtilis **Δ***degU*	Em^R*, B. subtilis* 168 *degU::erm*	[7]
B. subtilis degU32	Km^R*, B. subtilis* 168 *degU32*(Hy)	[8]
B. subtilis degU146	Km^R*, B. subtilis* 168 *degU146*	[9]
B. subtilis **Δ***bacA*	BFA3228 (*bacA*::pMUTIN)	Gift from Rob Meima

B. subtilis **Δ***bacD*	BFA3230 (*bacD*::pMUTIN)	Gift from Rob Meima
S. epidermidis Tü3298	Bap- strain	[10]
Cutibacterium acnes	NCTC737	[11]
S. thermophilus	CNRZ302	Gift from Luiza Morawska
B. subtilis ATCC 6633	*Bacillus subtilis* subsp. *spizizenii* strain ATCC 6633	[12]
B. subtilis NCIB 3610	Undomesticated strain. *Bacillus subtilis* NCIB 3610 is a derivative of Marburg with genomic similarity to *B. subtilis* 168.	[13]
B. subtilis PY79	*B. subtilis* strain PY79 is a prototrophic laboratory strain	[14]

Table S2. Oligonucleotides used in this study

Name	Sequence
27F	5’ AGAGTTTGATCMTGGCTCAG 3’
1492R	5’ CGGTTACCTTGTTACGACTT- 3’

Supplementary References

1. Kunst, F. *et al.* The complete genome sequence of the gram-positive bacterium *Bacillus subtilis*. *Nature.* **390**, 249–256 (1997). https://doi.org/10.1038/36786

2. Gasson, M. J. Plasmid complements of *Streptococcus lactis* NCDO 712 and other lactic streptococci after protoplast-induced curing. *J. Bacteriol.* **154**, 1-9 (1983).

3. Veening, J. W. *et al.* Transient heterogeneity in extracellular protease production by *Bacillus subtilis*. *Mol. Syst. Biol.* **4**, 184 (2008). doi:10.1038/msb.2008.18

4. Verhamme, D. T., Murray, E. J. & Stanley-Wall, N. R. DegU and Spo0A jointly control transcription of two loci required for complex colony development by *Bacillus subtilis*. *J. Bacteriol.* **191**, 100-108 (2009). doi:10.1128/JB.01236-08

5. Smits, W. K. *et al.* Stripping *Bacillus*: ComK auto-stimulation is responsible for the bistable response in competence development. *Mol. Microbiol.* **56**, 604-614 (2005). doi:10.1111/j.1365-2958.2005.04488.x

6. Diethmaier, C. *et al.* A novel factor controlling bistability in *Bacillus subtilis*: The Ymdb protein affects flagellin expression and biofilm formation. *J. Bacteriol. 193*, 5997-6007 (2011). doi:10.1128/JB.05360-11

7. Dartois, V., Débarbouillé, M., Kunst, F. & Rapoport, G. Characterization of a novel member of the DegS-DegU regulon affected by salt stress in *Bacillus subtilis. J. Bacteriol.* **180**, 1855-1861 (1998). doi:10.1128/jb.180.7.1855-1861.1998

8. Kunst, F., Msadek, T., Bignon, J. & Rapoport, G. The DegS/DegU and ComP/ComA two-component systems are part of a network controlling degradative enzyme synthesis and competence in *Bacillus subtilis*. *Research in Microbiology***.** **145**, 393-402 (1994). doi:10.1016/0923-2508(94)90087-6

9. Dahl, M. K., Msadek, T., Kunst, F. & Rapoport, G. Mutational analysis of the

Bacillus subtilis DegU regulator and its phosphorylation by the DegS protein kinase. *J. Bacteriol.* **173**, 2539-2547 (1991). doi:10.1128/jb.173.8.2539-2547.1991

10. Kies, S., Otto, M., Vuong, C. & Götz, F. Identification of the *sigB* operon in *Staphylococcus epidermidis*: Construction and characterization of a *sigB* deletion mutant. *Infect. Immun.* **69**, 7933-7936 (2001). doi:10.1128/IAI.69.12.7933-7936.2001

11. Scholz, C. F. P. & Kilian, M. The natural history of cutaneous propionibacteria, and reclassification of selected species within the genus *Propionibacterium* to the proposed novel genera *Acidipropionibacterium* gen. nov., *Cutibacterium* gen. nov. and *Pseudopropionibacterium* gen. nov. *Int. J. Syst. Evol. Microbiol.* **66**, 4422-4432 (2016). doi:10.1099/ijsem.0.001367

12. Waleh, N. S. & Ingraham, J. L. Pyrimidine ribonucleoside monophosphokinase and the mode of RNA turnover in *Bacillus subtilis*. *Arch. Microbiol.* **110**, 49-54 (1976). doi:10.1007/BF00416968

13. Nye, T. M., Schroeder, J. W., Kearns, D. B. & Simmons, L. A. Complete genome sequence of undomesticated *Bacillus subtilis* strain NCIB 3610. *Genome Announc.* **5**, e00364-17 (2017). doi:10.1128/genomeA.00364-17

14. Schroeder, J. W. & Simmons, L. A. Complete genome sequence of *Bacillus subtilis* strain PY79. *Genome Announc.* **1**, e01085-13 (2013). doi:10.1128/genomeA.01085-13
