# Supplementary material for: Impact of spatial proximity on territoriality among human skin bacteria
Source: NPJ Biofilms Microbiomes. 2020 Aug 6;6:30. doi: 10.1038/s41522-020-00140-0 (PMC7413532; doi:10.1038/s41522-020-00140-0)
Supplement: Supplementary file 2 — Reporting Summary [file 41522_2020_140_MOESM2_ESM.pdf]

nature research

Corresponding author(s): Oscar P. Kuipers

Last updated by author(s): 10th July 2020

Reporting Summary

Nature Research wishes to improve the reproducibility of the work that we publish. This form provides structure for consistency and transparency in reporting. For further information on Nature Research policies, see our Editorial Policies and the Editorial Policy Checklist.

Statistics

For all statistical analyses, confirm that the following items are present in the figure legend, table legend, main text, or Methods section.

n/a	Confirmed	
☐	☒	The exact sample size (*n*) for each experimental group/condition, given as a discrete number and unit of measurement
☐	☒	A statement on whether measurements were taken from distinct samples or whether the same sample was measured repeatedly
☒	☐	The statistical test(s) used AND whether they are one- or two-sided<br>*Only common tests should be described solely by name; describe more complex techniques in the Methods section.*
☒	☐	A description of all covariates tested
☒	☐	A description of any assumptions or corrections, such as tests of normality and adjustment for multiple comparisons
☐	☒	A full description of the statistical parameters including central tendency (e.g. means) or other basic estimates (e.g. regression coefficient) AND variation (e.g. standard deviation) or associated estimates of uncertainty (e.g. confidence intervals)
☒	☐	For null hypothesis testing, the test statistic (e.g. *F*, *t*, *r*) with confidence intervals, effect sizes, degrees of freedom and *P* value noted<br>*Give P values as exact values whenever suitable.*
☒	☐	For Bayesian analysis, information on the choice of priors and Markov chain Monte Carlo settings
☒	☐	For hierarchical and complex designs, identification of the appropriate level for tests and full reporting of outcomes
☒	☐	Estimates of effect sizes (e.g. Cohen's *d*, Pearson's *r*), indicating how they were calculated

Our web collection on statistics for biologists contains articles on many of the points above.

Software and code

Policy information about availability of computer code

Data collection	SoftWoRx 3.6.0 (Applied precision), FACSDiva Software 5.0.3 (BD Biosciences).
Data analysis	Graphpad Prism 8, breseq, Clustal Omega 1.2.2, FlowJo, Fiji 1.51d, ImageJ 1.52

For manuscripts utilizing custom algorithms or software that are central to the research but not yet described in published literature, software must be made available to editors and reviewers. We strongly encourage code deposition in a community repository (e.g. GitHub). See the Nature Research guidelines for submitting code & software for further information.

Data

Policy information about availability of data

All manuscripts must include a data availability statement. This statement should provide the following information, where applicable:

- Accession codes, unique identifiers, or web links for publicly available datasets
- A list of figures that have associated raw data
- A description of any restrictions on data availability

The S. epidermidis JH, S-1 and S-3 genomes were submitted to NCBI and are publicly available in the following accession codes: JAAUOD000000000 (JH; WT), JABTXG000000000 (S-1) and JABTXF000000000 (S-3).
Data supporting the findings of this work are available within the paper and its Supplementary Information files. All other data are available from the corresponding author on request.

Field-specific reporting

Please select the one below that is the best fit for your research. If you are not sure, read the appropriate sections before making your selection.

☒ Life sciences ☐ Behavioural & social sciences ☐ Ecological, evolutionary & environmental sciences

For a reference copy of the document with all sections, see nature.com/documents/nr-reporting-summary-flat.pdf

Life sciences study design

All studies must disclose on these points even when the disclosure is negative.

Sample size	No sample size was calculated. Sample size varied from a minimum of 3 independent experiments for interaction assays, plate reader assays and microscopy observations, whereas flow cytometry was performed with 30,000 cells. All sample sizes are indicated in the text or figure legends. Bacterial growth measurements are averages of three independent cultures. All replicates showed similar results.
Data exclusions	No data were excluded.
Replication	Bacterial growth measurements are averages of three independent cultures. All replicates showed similar results. Where possible, positive and negative controls for interaction assays were used to be sure of the accuracy of data and experimental conditions.
Randomization	Randomization was not necessary as samples were not allocated to groups.
Blinding	Blinding was not necessary as samples were not allocated to groups.

Reporting for specific materials, systems and methods

We require information from authors about some types of materials, experimental systems and methods used in many studies. Here, indicate whether each material, system or method listed is relevant to your study. If you are not sure if a list item applies to your research, read the appropriate section before selecting a response.

Materials & experimental systems

n/a	Involved in the study	
☒	☐	Antibodies
☒	☐	Eukaryotic cell lines
☒	☐	Palaeontology and archaeology
☒	☐	Animals and other organisms
☒	☐	Human research participants
☒	☐	Clinical data
☒	☐	Dual use research of concern

Methods

n/a	Involved in the study	
☒	☐	ChIP-seq
☐	☒	Flow cytometry
☒	☐	MRI-based neuroimaging

Flow Cytometry

Plots

Confirm that:

☒ The axis labels state the marker and fluorochrome used (e.g. CD4-FITC).

☒ The axis scales are clearly visible. Include numbers along axes only for bottom left plot of group (a 'group' is an analysis of identical markers).

☒ All plots are contour plots with outliers or pseudocolor plots.

☒ A numerical value for number of cells or percentage (with statistics) is provided.

Methodology

Sample preparation	B. subtilis strains were grown on interaction assays with S. epidermidis in CDM as described above. Biofilms were scraped from the CDM-agar surface and resuspended in 800 µL PBS. The biofilms were disrupted by repetitive passes through a 23G needle. Cells were subjected to a mild sonication to obtain a preparation of single cells.
Instrument	FACS Canto flow cytometer (BD Biosciences, CA, USA)
Software	FACSDiva 5.0.3 , FlowJo

Cell population abundance	The GFP-signal was recorded in 30,000 events
Gating strategy	A threshold for the FSC and SCC parameters was set (200 in both) in the FACS Canto flow cytometer (BD Biosciences, CA, USA) to remove all the events that do not correspond to cells. The GFP-signal at all the measured cells was recorded in 10,000 events and used for downstream analysis (named ungated events in the corresponding figures). GFP-signal measurements were obtained with a FACS Canto flow cytometer (BD Biosciences, CA, USA) using a 488 nm argon laser.

☒ Tick this box to confirm that a figure exemplifying the gating strategy is provided in the Supplementary Information.
